# Gut microbiota dysbiosis aggravates liver injury in acute-on-chronic liver failure through D-lactate accumulation

**DOI:** 10.3389/fmicb.2026.1851153

**Published:** 2026-09-10

**Authors:** Zhiwu Zeng, Lining Xu, Mingqing Yang, Weiyu Wang

**Affiliations:** 1 Department of General Surgery, Shenzhen Bao'an District Songgang People's Hospital, Shenzhen, China; 2 Department of General Surgery, The Second Medical Center & National Clinical Research Center for Geriatric Diseases, Chinese PLA General Hospital, Beijing, China; 3 Medical Experimental Center, Union Hospital, Tongji Medical College, Huazhong University of Science and Technology, Wuhan, China; 4 Zhongnan Hospital of Wuhan University, Wuhan, China; 5 Institute of Hepatobiliary Diseases of Wuhan University, Wuhan, China; 6 Transplant Center of Wuhan University, Wuhan, China; 7 National Quality Control Center for Donated Organ Procurement, Wuhan, China; 8 Hubei Key Laboratory of Medical Technology on Transplantation, Wuhan, China

**Keywords:** acute-on-chronic liver failure, gut microbiota, lactic acid, *Lactobacillus*, liver injury

## Abstract

**Introduction:**

Acute-on-chronic liver failure (ACLF) is a life-threatening condition arising from the abrupt worsening of pre-existing chronic liver disease, typically accompanied by multi-organ failure and markedly elevated short-term mortality. Gut microbiota dysbiosis is frequently observed in patients with ACLF, yet the mechanisms by which microbial alterations contribute to disease progression remain unclear. Here, we investigated whether gut microbiota dysbiosis could exacerbate liver injury in ACLF and explored the underlying mechanisms.

**Methods:**

The correlation between serum lactic acid levels and liver injury in patients with ACLF was analyzed. ACLF model in SD rats was established to investigate the effects of lactate transporter inhibitor on liver injury. 16S rRNA sequencing was performed to characterize alterations in the gut microbiota of ACLF rats, and correlations between serum lactate levels, fecal lactate levels and liver injury-related indicators were evaluated. A pseudo-germ-free ACLF rat model was established to determine whether gut microbiota dysbiosis could aggravate liver injury in ACLF rats.

**Results:**

Serum L-lactate and D-lactate levels were significantly positively correlated with ALT levels in patients with ACLF. Moreover, inhibiting lactic acid transport exacerbated liver injury in ACLF rats. The abundance of lactate-producing bacteria, particularly *Lactobacillus* and *Ligilactobacillus*, was significantly increased in the gut of ACLF rats, accompanied by elevated lactate levels in serum, liver tissues and feces. Correlation analysis revealed strong positive associations between serum lactic acid levels, fecal lactate levels and biochemical markers of liver injury, and between D-lactate levels and the abundance of lactate-producing taxa. Moreover, transplantation of fecal microbiota from conventional ACLF rats into pseudo-germ-free ACLF rats reduced microbial diversity, enriched lactate-producing genera, increased lactic acid levels in serum, liver tissues and feces, and exacerbated liver injury.

**Conclusion:**

Our results suggested that gut microbiota dysbiosis may contribute to ACLF progression, potentially through expansion of lactate-producing bacteria and subsequent D-lactic acid accumulation. Our results provided a conceptual basis for targeting microbial lactic acid metabolism as a therapeutic strategy for ACLF.

## 1 Introduction

Acute-on-chronic liver failure (ACLF) is a life-threatening clinical syndrome that arises in patients with an acute deterioration of underlying chronic liver disease (Trebicka et al., 2024). Although various regional criteria exist, major international societies agree that ACLF represents a distinct syndrome characterized by an acute hepatic insult in patients with underlying chronic liver disease, rapid deterioration of liver function, systemic inflammation, extrahepatic organ failures, and high short-term mortality (Chan et al., 2024; Luo et al., 2023). Emergency liver transplantation and comprehensive medical management remain the primary therapeutic options for ACLF (Br and Sarin, 2023). However, despite advances in artificial liver support systems and the standardization of intensive care, the mortality rate of ACLF remains unacceptably high (Perricone et al., 2023). A previous study showed that patients with acutely decompensated cirrhosis without ACLF have a 28-day mortality of less than 5%, whereas those who develop ACLF have a 28-day mortality exceeding 20% (Girish et al., 2013). Additionally, recent epidemiological data indicated that the 28-day and 90-day mortality rates of patients with ACLF are 38.6% and 45.8%, respectively (Liu et al., 2025). Therefore, these high mortality rates underscore the urgent need to refine our understanding of ACLF pathophysiology and to elucidate its underlying mechanisms, which are essential for improving prevention and therapeutic strategies.

ACLF has multifactorial etiologies; however, systemic inflammation and immune-metabolic dysregulation are widely recognized as key drivers of disease initiation and progression (Luo et al., 2023). Moreover, an increasing amount of evidence showed that ACLF patients exhibit an imbalance in the gut microbiota compared with healthy individuals, characterized by disrupted microbial diversity and compositional imbalance (Giuli et al., 2023; Trebicka et al., 2024). Therefore, some studies speculated that gut microbiota dysbiosis may be an important contributor to ACLF development (Kim et al., 2021; Zhang et al., 2018). Mechanically, disruption of the gut microbial ecosystem damages the gut barrier, allowing bacterial translocation across the damaged mucosa, which subsequently triggers systemic inflammation and immune dysfunction. Detailedly, compared with healthy individuals, ACLF patients exhibit a markedly increased intestinal ratio of cocci to bacilli (Wang K. et al., 2021). Moreover, *Lactobacillus casei paracasei* is significantly enriched during ACLF progression. Other associated study also reported that, compared with healthy individuals, the relative abundances of pathogenic bacteria such as *Escherichia, Collinsella*, and *Streptococcus* are significantly increased in the gut microbiota of patients with ACLF (Zeng et al., 2024). Noticeably, one study demonstrated that transplantation of fecal microbiota from healthy individuals significantly improved 28-day and 90-day survival rates, as well as clinical severity scores, in patients with severe alcoholic hepatitis-related ACLF (Sharma et al., 2022). Thus, restoring a healthy gut microbial ecosystem may represent a promising therapeutic strategy for ACLF.

*Lactobacillus*, a major genus of Gram-positive lactic acid-producing bacteria, plays a crucial role in maintaining intestinal homeostasis (Tao et al., 2025). Recently, accumulating evidence pointed that the relative abundance of *Lactobacillus* is significantly increased in the gut microbiota of patients with ACLF, suggesting that *Lactobacillus* may be positively associated with ACLF progression (Wang K. et al., 2021; Yao et al., 2021). One of the primary functions of *Lactobacillus* is the production of lactic acid, and the increased abundance of *Lactobacillus* in the gut of patients with ACLF indicates an enhanced generation of lactic acid (Lobeda et al., 2022). And more notably, some studies reported that lactic acid produced by *Lactobacillus* within the gut can traverse the gut epithelial barrier and enter the bloodstream, allowing systemic distribution to peripheral tissues (Oliphant and Allen-Vercoe, 2019; Remund et al., 2023). The liver is the principal organ responsible for lactic acid clearance, accounting for approximately 70% of systemic lactic acid removal in the human body (Yao et al., 2024). However, elevated lactic acid levels are strongly associated with the severity and progression of liver failure (Lin et al., 2023). A previous study showed that the concentration of lactic acid in ascitic fluid is significantly higher in patients with ACLF than in those with other chronic liver diseases, and ACLF patients with lactate levels exceeding 2 mmol/L exhibit markedly poorer clinical outcomes (Mani et al., 2021). Moreover, serum lactic acid level has also been identified as an independent risk factor of 90-day mortality in ACLF patients with concurrent infection (Li et al., 2023). Therefore, we hypothesized that lactic acid produced by *Lactobacillus* and other lactate-producing bacteria in the gut of patients with ACLF may traverse the intestinal barrier and enter the systemic circulation, thereby exacerbating disease severity. However, to date, no studies have directly investigated this possibility.

In this study, we have established a rat model of ACLF to investigate whether disordering of the gut microbiota exacerbates ACLF by increasing lactic acid levels. Our results could enrich current understanding of the pathogenic mechanisms underlying ACLF and provide a theoretical basis for the development of novel therapeutic strategies.

## 2 Materials and methods

### 2.1 Chemicals and reagents

CCl_4_ (Cat No. 10006464) was purchased from Sinopharm Chemical Reagent Co., Ltd. (Shanghai, China). Lactate transporter inhibitor (AR-C155858; Cat No. S85548), d-galactosamine (D-Gal; Cat No. S11019), metronidazole (Cat No. S17079), ampicillin (Cat No. S17018) and vancomycin hydrochloride (Cat No. S17059) were purchased from Shanghai YuanYe Biotechnology Co., Ltd. (Shanghai, China). Neomycin sulfate (Cat No. ST2528), L-Lactate assay kit (Cat No. S0208S) and D-Lactate assay kit (Cat No. S0204S) were purchased from Beyotime (Shanghai, China). Masson assay kit (Cat No. BA4079B) was purchased from BASO (Zhuhai, China). Alanine aminotransferase (ALT) assay kit, aspartate aminotransferase (AST) assay kit and alkaline phosphatase (ALP) assay kit were purchased from Rayto (Shenzhen, China). Total bilirubin (T-BIL) assay kit was purchased from Changchun Huili Biotech Co., Ltd. (Changchun, China). Primary antibodies against MCT1 (Cat No. 20139-1-AP) and MCT2 (Cat No. 20355-1-AP) and second antibody HRP-conjugated Goat Anti-Rabbit IgG(H+L; Cat No. SA00001-2) were purchased from Wuhan Sanying Biotechnology Co., Ltd. (Wuhan, China). IL-1β ELISA Kit (LJS-R-001), IL-6 ELISA Kit (LJS-R-004), TNF-α ELISA Kit (LJS-R-006) and primary antibody against β-actin (Cat No. LJS-A-7018) was purchased from Wuhan Lingjiesi Biotechnology Co., Ltd. (Wuhan, China). VAMNE Stool/Soil DNA Extraction Kit (Cat. No. DM401-C3-P2) was purchased from Vazyme (Nanjing, China).

### 2.2 Human samples collection

Serum samples from patients with ACLF and chronic liver disease (CLD) were obtained from individuals diagnosed and treated from June 2025 to December 2025 in the Zhongnan Hospital of Wuhan University. All patients in both groups had chronic hepatitis B (CHB). CHB was diagnosed based on hepatitis B surface antigen positivity and/or detectable hepatitis B virus DNA (HBV-DNA) for at least 6 months (Hou et al., 2017). Hepatitis B virus-related ACLF (HBV-ACLF) was diagnosed according to the COSSH-HBV-ACLF criteria and the Chinese guidelines (Wu et al., 2018). Briefly, patients with CHB, with or without cirrhosis, were diagnosed with HBV-ACLF when they presented with total bilirubin (TBil) ≥12 mg/dL and an international normalized ratio (INR) ≥1.5. Patients with CHB who did not meet the diagnostic criteria for HBV-ACLF were included in the CLD group. The exclusion criteria were age < 18 years, pregnancy, pre-existing renal disease, hepatocellular carcinoma, extrahepatic malignancies, other serious systemic diseases, and concomitant liver diseases other than CHB, including autoimmune liver disease, schistosomiasis-related liver disease, and alcohol-related liver disease. In total, this study included 9 patients who met the diagnostic criteria for HBV-ACLF and 9 patients who met the diagnostic criteria for CLD.

The formula for the model for end-stage liver disease (MELD) score was as follows: MELD score = 3.78 × ln [Total bilirubin (mg/dL)] + 11.2 × ln (INR) + 9.57 × ln [Creatinine (mg/dL)] + 6.43 (Kamath et al., 2007). The sex, age and MELD score of the patients are presented in Supplementary Table S1.

Our study complies with the Declaration of Helsinki. Informed consent (oral and written) was obtained from all participants. Clinical experiments were approved by the Ethics Committee of Zhongnan Hospital of Wuhan University (Approved No. 2025141K).

### 2.3 Animals

Male Sprague-Dawley (SD) rats (6–8 weeks of age, 180–220 g) were obtained from Spefu (Beijing) Biotechnology Co., Ltd. (Beijing, China). Animals were housed in a temperature-controlled environment (22 ± 2 °C) with 55 ± 5% relative humidity under a 12-h light/dark cycle, with free access to food and water. All experimental procedures involving animals were approved by the Institutional Animal Care and Use Committee of Zhongnan Hospital of Wuhan University (Approval no. ZN2024235). The animal experiments followed the ARRIVE 2.0 guidelines for reporting animal experiments (Percie du Sert et al., 2020).

### 2.4 Animals grouping and treating

The animal experiments in this study consisted of two independent parts. The grouping and treatment procedures for each part were described separately in detail below.

**Figure 1:**
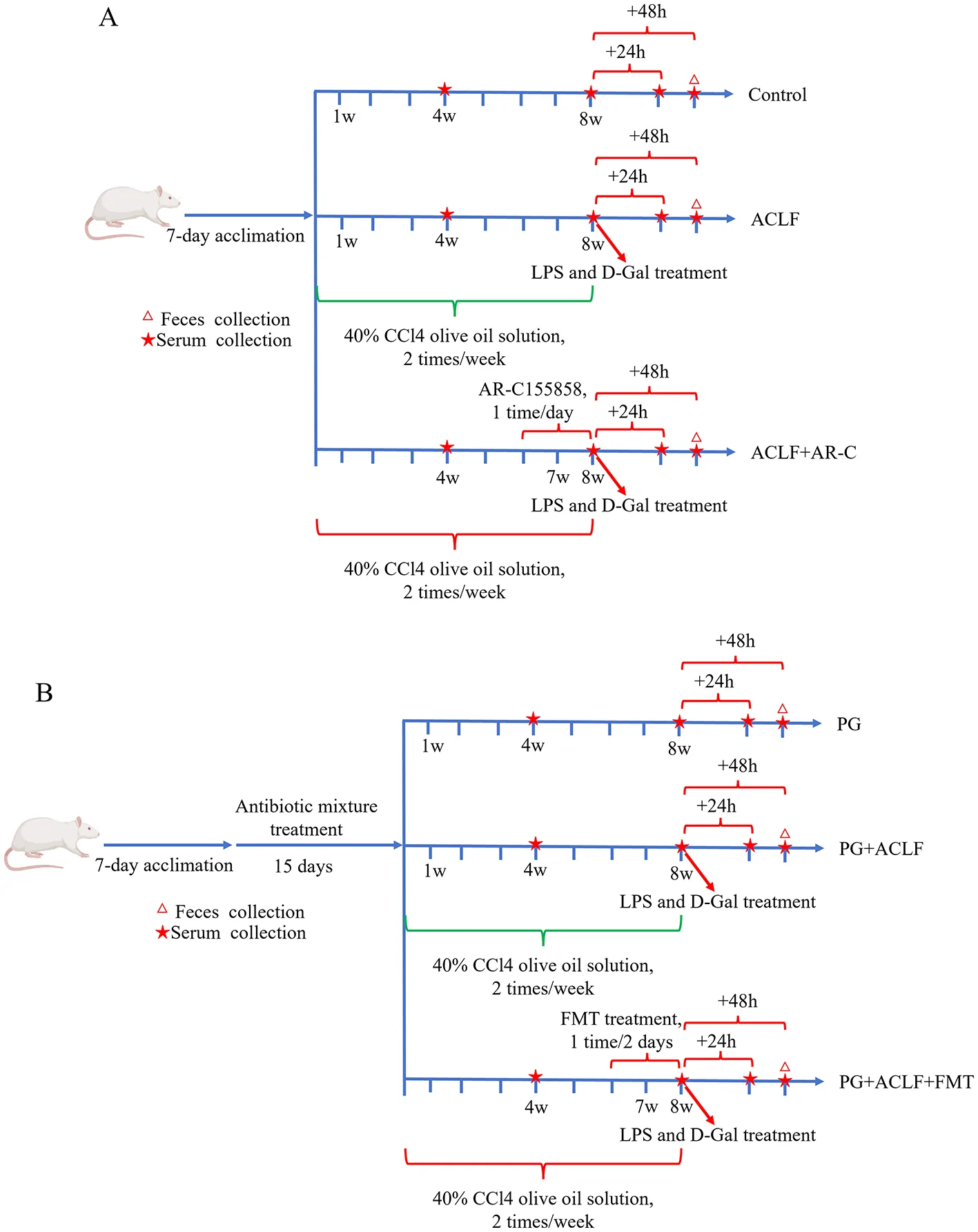
Flow chart of the study. **(A)** The flow chart of the Part 1. **(B)** The flow chart of the Part 2.

Part 1:

After a 7-day acclimation period, male SD rats were randomly assigned to 3 groups (*n* = 6 per group): Control group (Control), ACLF model group (ACLF) and ACLF + lactate transporter inhibitor group (ACLF+AR-C). The ACLF model was established according to the method described by Liang et al. (2023). Briefly, rats received intraperitoneal injections of 40% CCl_4_ olive oil solution (1.5 mL/kg), 2 times per week. After 8 weeks of CCl_4_ administration, acute liver injury was induced by intraperitoneal injection of LPS (100 μg/kg) combined with D-Gal (400 mg/kg), thereby generating the ACLF model. For the ACLF+AR-C, rats underwent the same ACLF induction protocol, and lactate transporter inhibitor was administered once daily during weeks 7 and 8. According to previous research, AR-C155858 was used as the lactate transporter inhibitor in this study (Richiardone et al., 2024). AR-C155858 was administered intraperitoneally at a dose of 3 mg/kg. Rats in the Control group received an equal volume of normal saline by intraperitoneal injection at the corresponding time points and were not subjected to ACLF induction or AR-C155858 intervention. Rat blood samples were collected at 24 h and 48 h after the acute attack. No rats died during the experimental period. Rats in each group were sacrificed 48 h after the acute attack. After anesthesia with 3% sodium pentobarbital (2 mL/kg, intraperitoneally), the rats were euthanized by cervical dislocation. Fecal and liver tissue samples were then collected for further analysis. Figure 1A showed the flow chart of the Part 1.

Part 2:

After a 7-day acclimation period, male SD rats were randomly assigned to 3 groups (*n* = 6 per group): the pseudo-germ-free group (PG), the pseudo-germ-free + ACLF model group (PG+ACLF), and the pseudo-germ-free + ACLF + fecal microbiota transplantation group (PG+ACLF+FMT). Pseudo-germ-free rats were generated according to previously described methods (Zheng et al., 2024; Zhong W. et al., 2022). Briefly, rats were provided drinking water containing vancomycin (0.5 g/L), neomycin sulfate (1 g/L), metronidazole (1 g/L), and ampicillin (1 g/L) for 15 consecutive days to deplete the intestinal microbiota. Rats in the PG group received the antibiotics mixture to establish the pseudo-germ-free condition but were not subjected to ACLF induction or FMT.

In the PG+ACLF, pseudo-germ-free rats received intraperitoneal injections of 40% CCl_4_ olive oil solution (1.5 mL/kg), twice weekly for 8 weeks. In the 8th week, a single intraperitoneal injection of LPS (100 μg/kg) was administered, followed 30 min later by D-Gal (400 mg/kg) to induce ACLF. In the PG+ACLF+FMT group, pseudo-germ-free ACLF rats received fecal microbiota transplantation by oral gavage using fecal microbiota suspensions prepared from conventionally raised ACLF model rats in Part 1. At the end of Part 1, fresh fecal samples were collected from conventionally raised ACLF model rats. A portion of the fecal samples was used for 16S rRNA sequencing to characterize the donor microbial composition, while the remaining samples were immediately processed to prepare fecal microbiota suspensions for oral gavage into pseudo-germ-free rats. Fecal microbiota suspensions were administered at a dose of 5 × 10^8^ CFU every 2 days during weeks 7–8. This dose and frequency were selected based on previously published FMT studies in rodent models and were intended to maintain microbial colonization during the intervention period (Sun et al., 2024; Zhang et al., 2022; Zhong Y. et al., 2022). At the end of week 8, the same LPS (100 μg/kg) and D-Gal (400 mg/kg) challenge was performed to induce ACLF. Rat blood samples were collected at 24 h and 48 h after the acute attack. No rats died during the experimental period. Rats in each group were sacrificed 48 h after the acute attack. After anesthesia with 3% sodium pentobarbital (2 mL/kg, intraperitoneally), the rats were euthanized by cervical dislocation. Fecal and liver tissue samples were then collected for further analysis. Figure 1B showed the flow chart of the Part 2.

No animals died during the experimental period, and no animals or biological samples were excluded from the subsequent analyses. All animals initially assigned to the experimental groups were included in the final statistical analysis. A *post hoc* power analysis was performed using G^*^Power software (version 3.1.9.7; Heinrich-Heine-Universität Düsseldorf, Düsseldorf, Germany). For the one-way ANOVA, the parameters included an α level of 0.05, 3 experimental groups, a total sample size of 18 rats (*n* = 6 per group), and a large effect size of f = 0.40 according to Cohen's criteria. The calculated statistical power (1 – β) was 0.26.

### 2.5 Hematoxylin-eosin (HE) staining

Rat liver tissues were fixed in 4% paraformaldehyde for at least 24 h. After fixation, the tissues were dehydrated through a graded ethanol series and embedded in paraffin. Paraffin blocks were sectioned into 4-μm slices using a microtome and mounted onto glass slides. Following deparaffinization and rehydration through graded ethanol and water, sections were stained with hematoxylin and subsequently with eosin. After dehydration and clearing with graded ethanol and xylene, the sections were mounted with neutral resin. Histological images were captured using an optical microscope.

### 2.6 Masson staining

Paraffin-embedded tissue blocks were sectioned into 4 μm slices using a microtome and mounted onto glass slides. Sections were deparaffinized and rehydrated using xylene, a graded ethanol series and water. Nuclei were stained with Weigert's iron hematoxylin (solution A/B mixture) for 5 min, followed by rinsing under running water for 10 min. Then, sections were stained with Biebrich scarlet-acid fuchsin solution for 5 min and washed again under running water for 10 min. Differentiation was performed in phosphomolybdic acid solution for approximately 5 min, after which excess reagent was gently removed without washing. Sections were immediately counterstained with aniline blue for 5 min. After dehydration through graded ethanol and clearing in xylene, sections were mounted with neutral resin. Histopathological changes were observed and imaged using a light microscope.

### 2.7 Serum biochemical analysis and inflammatory factor detection

Human and rat blood samples were collected for serum biochemical analysis. Blood samples were centrifuged at 1,000 × g for 10 min, and the supernatant was collected as serum. Serum levels of ALT, AST, ALP, and T-BIL were measured using the corresponding commercial assay kits. Prepared serum samples were loaded into an automated biochemical analyzer (Chemray 420; Rayto, Shenzhen, China) for fully automated detection. Serum levels of IL-1β, IL-6 and TNF-α in rats were measured using enzyme-linked immunosorbent assay kits according to the manufacturers' instructions.

### 2.8 Lactic acid detection

Lactic acid concentrations in human serum, rat serum, liver tissues and feces were measured using corresponding assay kits.

Lactate levels in human and rat serum were measured using a WST-8-based colorimetric assay. Briefly, 50 μL of serum sample was added to each sample well of a 96-well plate, followed by 50 μL of WST-8 chromogenic working solution. The mixture was incubated at 37 °C for 30 min in the dark, and absorbance was measured at 450 nm. Lactate concentrations were calculated from a standard curve.

For the determination of lactic acid in rat liver, liver tissues were first homogenized. Briefly, 10 mg of liver tissue was mixed with 100 μL of BeyoLysis™ Buffer A for Metabolic Assays and homogenized at low temperature using a tissue grinder. The homogenate was centrifuged at 12,000 × g for 5 min at 4 °C, and the supernatant was collected for subsequent analysis. For each assay, 50 μL of the tissue supernatant was added to a 96-well plate, followed by 50 μL of WST-8 working solution. After gentle mixing, samples were incubated at 37 °C in the dark for 30 min. Absorbance at 450 nm was then measured using a microplate reader.

Fecal lactate levels were measured using a commercial lactate assay kit according to the manufacturer's instructions. Briefly, fecal samples were homogenized after 10-fold dilution by using PBS solution and centrifuged to collect the supernatant. Then, 50 μL of fecal homogenate supernatant was added to each well of a 96-well plate, followed by 50 μL of WST-8 working solution. After gentle mixing, the plate was incubated at 37 °C for 30 min in the dark. The absorbance was measured at 450 nm, and lactate concentrations in the supernatants were calculated according to the standard curve.

### 2.9 16S rRNA sequencing

Rat fecal samples were collected for 16S rRNA sequencing to analyze gut microbiota composition. Microbial DNA was extracted from fecal samples using the VAMNE Stool/Soil DNA Extraction Kit according to the manufacturer's instructions. DNA quality was assessed using a NanoDrop spectrophotometer and 1.8% agarose gel electrophoresis. The full-length 16S rRNA gene was amplified using the primers 5′-AGRGTTTGATYNTGGCTCAG-3′ and 5′-TASGGHTACCTTGTTASGACTT-3′. Purified amplicons were used to construct SMRTbell libraries and sequenced on the PacBio Sequel II platform, generating approximately 50,000 circular consensus sequencing (CCS) reads per sample.

Raw BAM files were processed using SMRT Link software (version 8.0) with a minimum of five passes and a predicted accuracy of ≥0.90. CCS reads were demultiplexed using lima (version 1.7.0), and primer sequences were removed using Cutadapt (version 2.7). Reads lacking complete primer sequences or falling outside the expected length range of 1,200–1,650 bp were discarded. Chimeric sequences were identified and removed using UCHIME (version 8.1). High-quality sequences were clustered into operational taxonomic units (OTUs) at 97% sequence similarity using USEARCH (version 10.0), and singleton OTUs represented by only one sequence across the entire dataset were excluded. Taxonomic annotation was performed using the naïve Bayes classifier implemented in the QIIME 2 feature-classifier plugin against the SILVA database (release 138), with the NCBI nucleotide database downloaded in 2024 used for supplementary taxonomic verification. Before diversity analyses, the OTU table was rarefied to 40,000 sequences per sample using the qiime feature-table rarefy function in QIIME 2 (version 2023.7). Rarefaction curves were generated to evaluate sequencing-depth sufficiency. The numbers of Raw CCS and Clean CCS reads and the sequence-quality statistics for each sample are provided in Supplementary Table S2.

Taxonomic annotation was performed against the SILVA release 138 database (http://www.arb-silva.de), with the NCBI nucleotide database used for supplementary assignment (ftp://ftp.ncbi.nih.gov/blast/db/). Rarefaction curves approached a plateau at approximately 40,000 sequences per sample, indicating that the sequencing depth was sufficient to capture most of the detectable microbial richness. The species accumulation curve also gradually leveled off as the number of samples increased, suggesting that the included samples provided reasonable coverage of the microbial community. 16S rRNA sequencing was performed by Wuhan Lingsi Biotechnology Co., Ltd. (Wuhan, China).

### 2.10 Western blot (WB)

Total protein from rat liver tissues was extracted using RIPA lysis buffer. Total protein (40 μg) was separated by sodium dodecyl sulfate (SDS)-polyacrylamide gel electrophoresis (PAGE). Subsequently, target proteins were transferred onto PVDF membranes using a wet-transfer system. After washing with TBST, membranes were blocked with 5% skim milk at 25 °C for 30 min. Then, membranes were incubated overnight at 4 °C with primary antibodies against MCT1 (1:5,000), MCT2 (1:1,000), and β-actin (1:10,000). Following 3 washes with TBST (5 min each), membranes were incubated with HRP-conjugated goat anti-rabbit IgG (H+L) secondary antibody (1:10,000) at 25 °C for 60 min. After TBST washes (3 times, 5 min each), protein bands were visualized using an ECL detection kit and imaged with a chemiluminescence imaging system. Band intensities were quantified using ImageJ software.

### 2.11 Statistical analysis

The normality of continuous clinical variables was assessed using the Shapiro-Wilk test, and homogeneity of variance was evaluated using an F test. ALT, AST, and ALP values, for which at least one group did not meet the assumption of normality, are presented as the median with interquartile range (IQR) and were compared between the ACLF and chronic liver disease groups using a two-sided Mann-Whitney test. T-BIL, the AST/ALT ratio, L-lactate and D-lactate values were normally distributed and showed equal variances; these data are presented as the mean ± standard deviation (SD) and were compared using a two-sided unpaired Student's *t*-test. In patients with ACLF, the associations of serum D-lactate and L-lactate concentrations with serum ALT levels were assessed separately using two-sided Spearman's rank correlation analysis because of the small sample size and non-normal distribution of the data. Spearman's correlation coefficient (*r*) and exact *P* values were reported. All available observations were included in the analysis, and no values were excluded solely on the basis of their magnitude.

Indicators of liver injury, inflammatory cytokine levels, lactate concentrations and protein expression levels in rats were normally distributed. All data are presented as the mean ± SD. Comparisons among the three groups were performed using one-way analysis of variance (ANOVA), followed by Tukey's *post hoc* multiple-comparisons test. The associations of serum and fecal lactate concentrations with serum ALT levels were assessed using Pearson's correlation analysis. Pearson's correlation coefficient (*r*), 95% confidence interval (CI), and exact *P* value were reported.

α-diversity indices were calculated separately for each fecal sample. Extreme values were examined for potential technical or data-entry errors, and valid observations were not excluded solely on the basis of their magnitude. Given the small sample size and the presence of extreme values, alpha-diversity data are presented as the median with interquartile range (IQR). Differences among the three groups were assessed using the Kruskal-Wallis test followed by Dunn's multiple-comparisons test. Individual data points are shown in the corresponding plots. β diversity was assessed using Bray–Curtis dissimilarity and visualized by principal coordinates analysis (PCoA). Differences in microbial community composition among the groups were evaluated using permutational multivariate analysis of variance (PERMANOVA) based on the Bray-Curtis distance matrix, with the pseudo-*F* statistic and *P* value calculated from 999 permutations. The association between fecal microbial relative abundance and serum D-lactate concentrations was assessed using two-sided Spearman's rank correlation analysis.

Statistical analyses and data visualization were performed with GraphPad Prism 8.0.1 (GraphPad Software, San Diego, CA, USA). No observations were excluded as outliers, and all available measurements were included in the statistical analyses in this study. Differences were considered statistically significant at *P* < 0.05.

## 3 Results

### 3.1 Serum lactic acid levels were associated with liver injury in patients with ACLF

**Figure 2:**
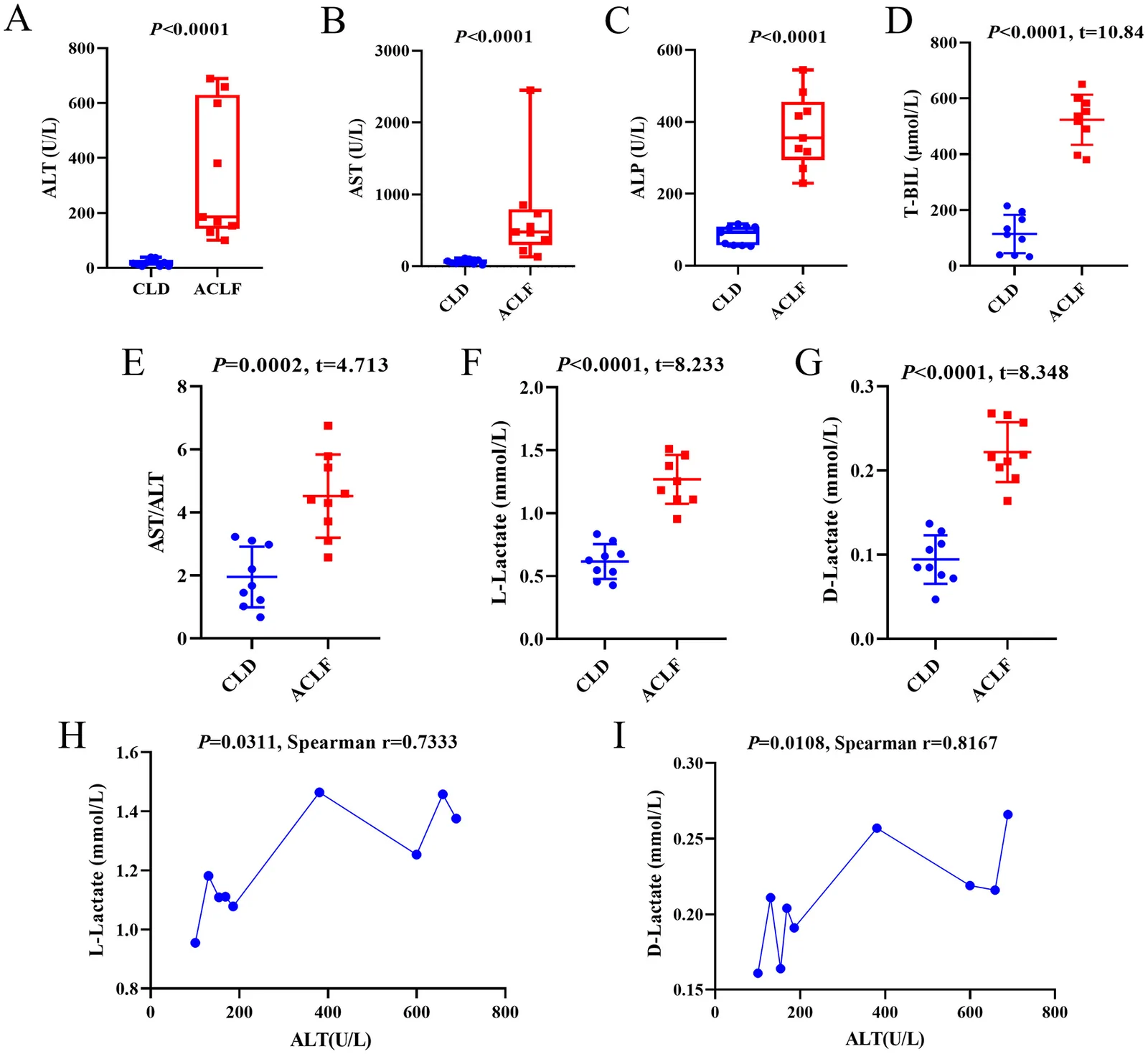
Serum lactic acid levels were associated with liver injury in patients with ACLF. **(A–D)** Serum levels of ALT **(A)**, AST **(B)**, ALP **(C)**, and T-BIL **(D)** in patients with ACLF and chronic liver disease. **(E)** Serum AST/ALT ratio in patients with ACLF and chronic liver disease. **(F, G)** Serum levels of L-lactate **(F)** and D-lactate **(G)** in patients with ACLF and chronic liver disease. **(H, I)** Spearman correlation analysis between serum L-lactate **(H)** and D-lactate **(I)** levels and ALT in patients with ACLF. *n* = 9 biological replicates per group. Differences in ALT, AST, and ALP levels between the two groups were assessed using a two-sided Mann-Whitney test. T-BIL, the AST/ALT ratio, L-lactate and D-lactate levels were compared using a two-sided unpaired Student's *t*-test.

Firstly, we have compared serum markers of liver injury (ALT, AST, ALP, and T-BIL), L-lactate and D-lactate levels between patients with ACLF and those with chronic liver disease. Serum biochemical analyses revealed that ALT, AST, ALP, and T-BIL levels were significantly higher in patients with ACLF than in those with chronic liver disease (*P* < 0.05; Figures 2A–D). Notably, the AST/ALT ratio was significantly higher in patients with ACLF than in those with chronic liver disease (*P* < 0.01; Figure 2E). Additionally, serum levels of both L-lactate and D-lactate were markedly elevated in patients with ACLF (*P* < 0.01; Figures 2F, G). Correlation analysis revealed significant positive correlations between serum L-lactate and D-lactate levels and ALT in patients with ACLF (*P* < 0.05; Figures 2H, I), indicating that higher circulating L-lactate and D-lactate levels may be associated with more severe liver injury in ACLF.

### 3.2 Inhibition of lactic acid transport exacerbated liver injury and systemic inflammation in the ACLF rat model

**Figure 3:**
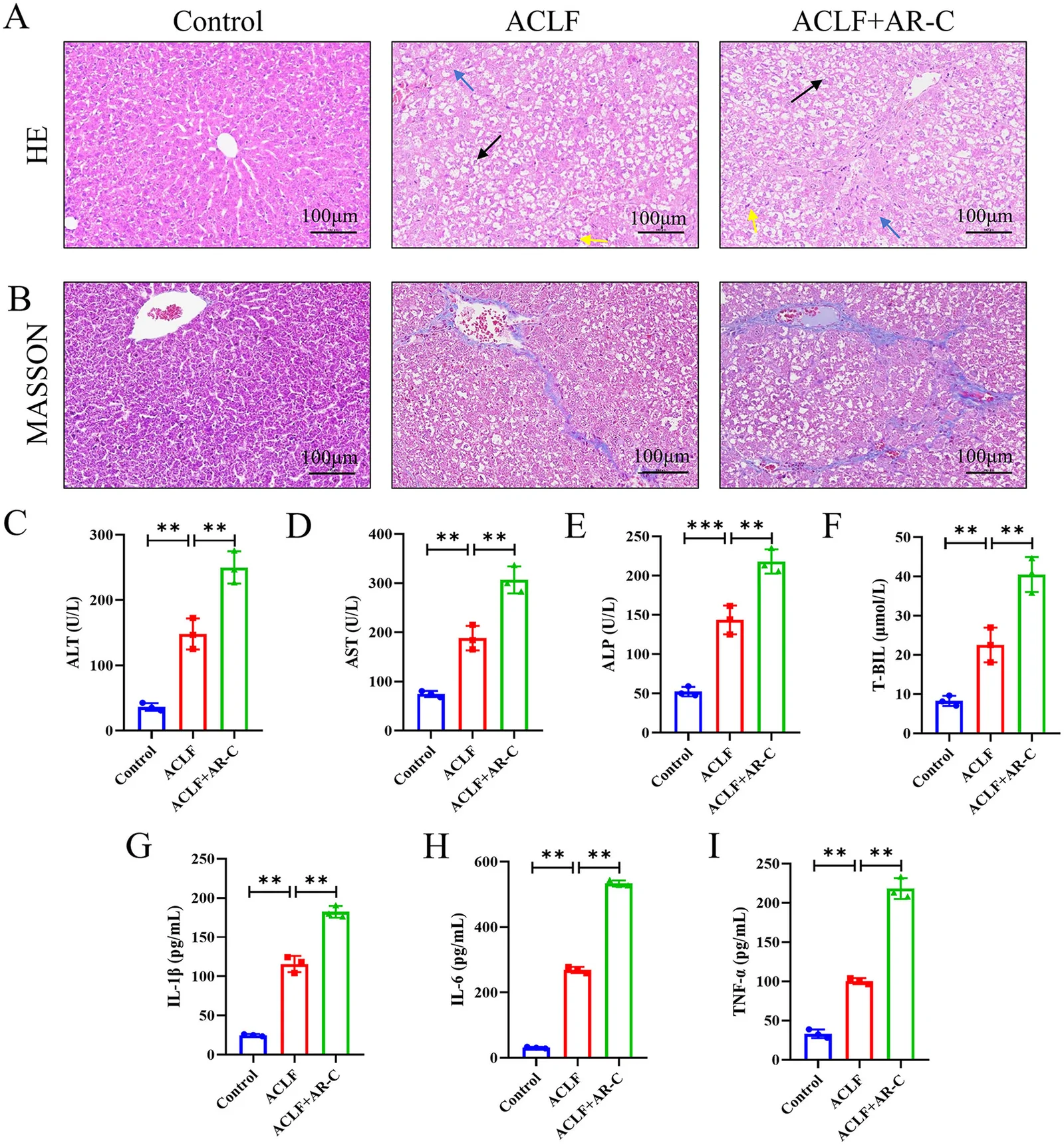
Assessment of liver injury and systemic inflammation in rats. **(A)** HE staining was used to observe histopathological alterations. **(B)** Masson staining was used to evaluate liver fibrosis. **(C–I)** Serum levels of ALT **(C)**, AST **(D)**, ALP **(E)**, T-BIL **(F)**, IL-1β **(G)**, IL-6 **(H)**, and TNF-α **(I)**. *n* = 3 biological replicates per group. Comparisons among the three groups were performed using one-way analysis of variance (ANOVA), followed by Tukey's *post hoc* multiple-comparisons test. **P < 0.01, ***P < 0.001.

HE staining (Figure 3A) revealed that liver tissues from the Control group exhibited intact morphological architecture, with uniformly distributed hepatic lobules, orderly hepatic cords, and regularly arranged hepatocytes, without apparent pathological alterations. In contrast, the ACLF group showed disrupted hepatic cord architecture, disorganized hepatocyte arrangement, marked hepatocellular swelling with cytoplasmic rarefaction, and pronounced connective tissue proliferation in the portal areas accompanied by inflammatory cell infiltration. Similar histopathological abnormalities were observed in the ACLF+AR-C group, including disordered hepatic plates, hepatocyte swelling, and cytoplasmic rarefaction, along with mild connective tissue proliferation in the portal region. Additionally, Masson staining showed increased collagen deposition in the liver tissues of ACLF rats, with more pronounced fibrotic changes observed in the ACLF+AR-C group, suggesting that lactate transport inhibition may be associated with more severe liver fibrosis under ACLF conditions (Figure 3B).

Serum biochemical assays revealed that serum levels of ALT, AST, ALP, and T-BIL were significantly higher in the ACLF group than in the Control group at 48 h after the acute attack (*P* < 0.01). Moreover, treatment with a lactate transport inhibitor further increased serum ALT, AST, ALP, and T-BIL levels in ACLF model rats (*P* < 0.01; Figures 3C–F). Consistently, serum levels of the inflammatory cytokines IL-1β, IL-6 and TNF-α were also markedly elevated in the ACLF group compared with the Control group at 48 h after the acute attack (*P* < 0.01). Lactate transport inhibitor treatment further enhanced the serum levels of IL-1β, IL-6 and TNF-α in ACLF model rats (*P* < 0.01; Figures 3G–I).

Collectively, these findings indicated that inhibition of lactate transport could exacerbate liver injury and systemic inflammation in the ACLF rat model.

### 3.3 ACLF disordered the gut microbiota composition in rats

**Figure 4:**
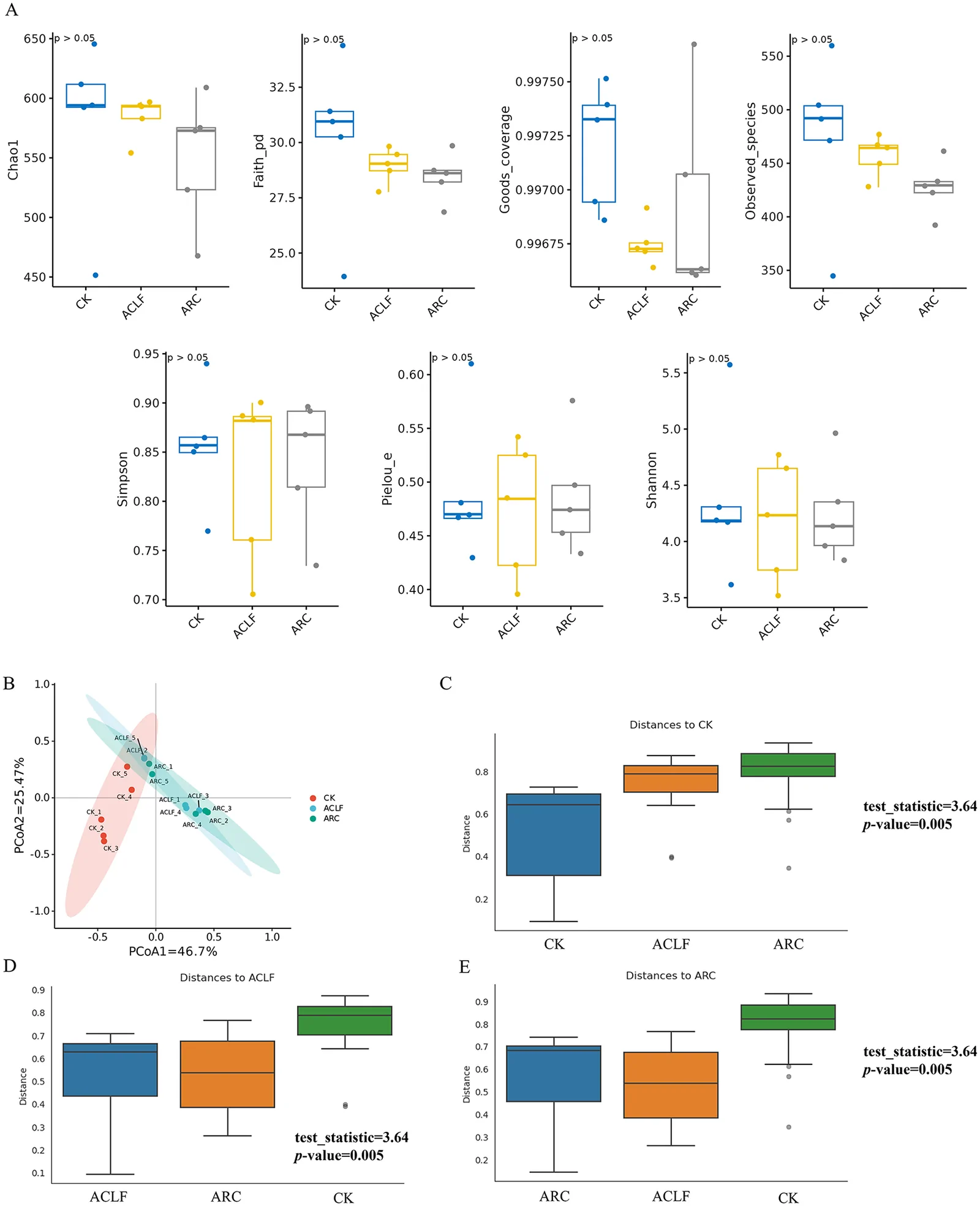
Analysis of gut microbial diversity. **(A)** Comparison of gut microbial α-diversity among the different groups. **(B)** PCoA of the gut microbiota based on Bray-Curtis distance. **(C)** β-diversity distances to the CK group based on Bray-Curtis dissimilarity. **(D)** β-diversity distances to the ACLF group based on Bray-Curtis dissimilarity. **(E)** β-diversity distances to the ARC group based on Bray-Curtis dissimilarity. *n* = 5 biological replicates per group. Differences in α-diversity indices among groups were assessed using the Kruskal-Wallis test followed by Dunn's multiple-comparisons test. Differences in β-diversity among groups were evaluated using permutational multivariate analysis of variance (PERMANOVA) based on Bray-Curtis dissimilarity, with the pseudo-F statistic used to assess statistical significance.

16S rRNA sequencing was employed to characterize differences in gut microbial composition among the experimental groups. Supplementary Table S3 summarized the number of microbial taxonomic units detected in each sample. At the phylum level, an average of 13 phyla were identified in the Control group, compared with 12 phyla in both the ACLF and ACLF+AR-C groups. At the genus level, the Control group exhibited an average of 245 genera, whereas the ACLF and ACLF+AR-C groups showed slightly reduced counts of 243 and 236 genera, respectively. Moreover, analysis of α-diversity revealed that compared with the control group, rats with ACLF showed trends toward lower Chao1, Faith's phylogenetic diversity, Good's coverage and observed species, together with higher Simpson, Shannon and Pielou's evenness indices; however, none of these differences reached statistical significance. Compared with the ACLF group, AR-C155858 treatment tended to reduce Chao1, Faith's phylogenetic diversity, Good's coverage, observed species, Simpson, Shannon and Pielou's evenness indices, although these changes were not statistically significant (Figure 4A). Supplementary Figure S1 shows the rarefaction curve, species accumulation curve, and rank abundance curve. Principal coordinate analysis (PCoA) based on Bray-Curtis distance showed a clear separation of microbial community structures among the groups (Figures 4B–E). In summary, the ACLF group showed a slight reduction in taxonomic counts at the phylum and genus levels compared with the Control group, suggesting a degree of community simplification and decreased microbial richness under ACLF conditions.

**Figure 5:**
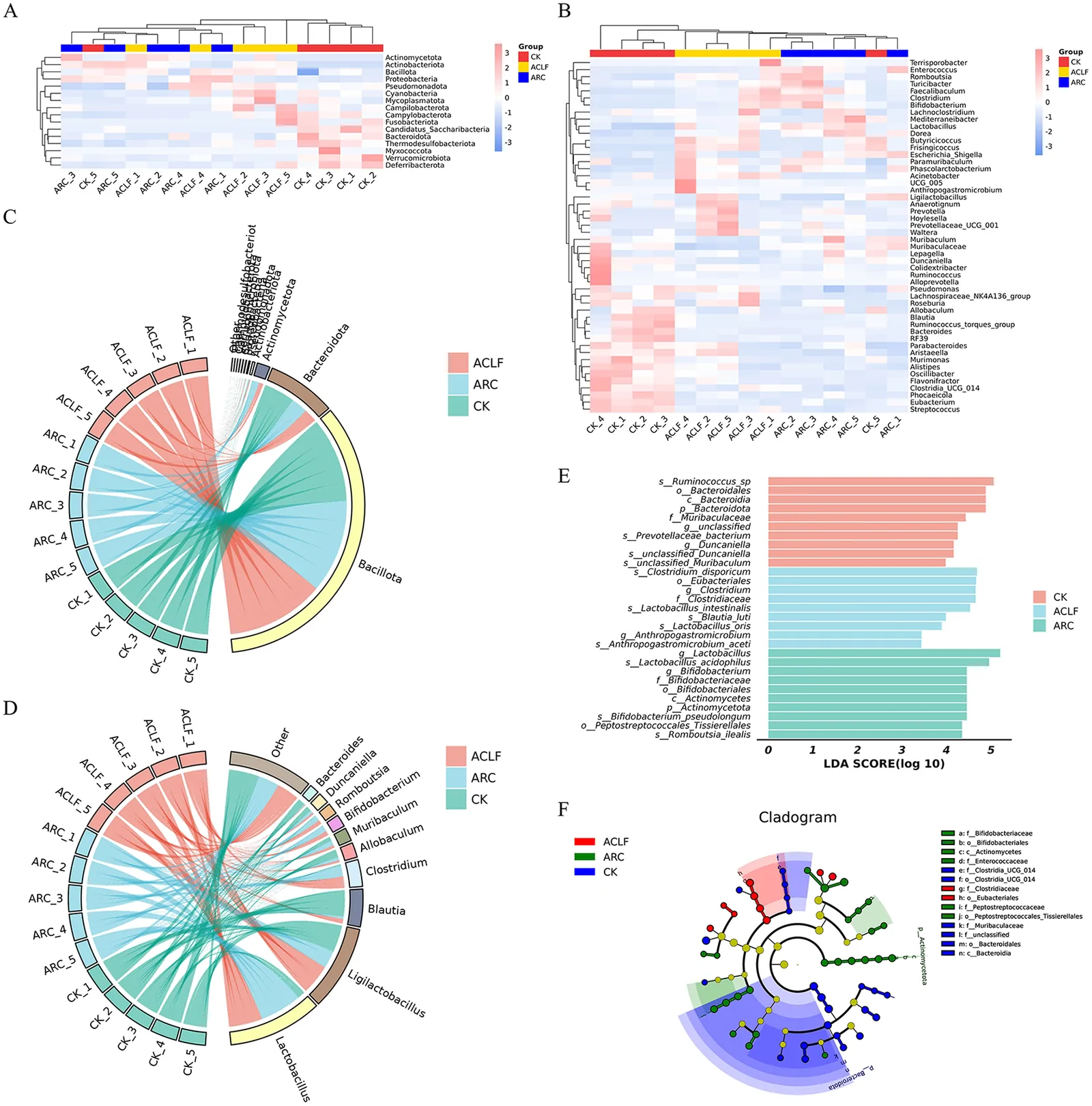
Differences in gut microbial composition and taxonomic biomarkers among groups. **(A)** Heatmap of differential bacterial abundance at the phylum level. **(B)** Heatmap of differential bacterial abundance at the genus level. **(C)** Analysis of dominant bacterial taxa at the phylum level. **(D)** Analysis of dominant bacterial taxa at the genus level. **(E)** LDA score bar plot. **(F)** Cladogram showing the taxonomic classification of the identified species. CK, Control group; ACLF, ACLF model group; ARC, ACLF + lactate transporter inhibitor group. *n* = 5 biological replicates per group.

Further, we have analyzed the effects of ACLF and lactate transport inhibition on the gut microbiota at both the phylum and genus levels in rats. At the phylum level, compared with the Control group, ACLF rats showed a marked decrease in the abundances of *Bacteroidota, Verrucomicrobiota*, and *Deferribacterota*, accompanied by significant increases in *Bacillota, Actinomycetota*, and *Actinobacteriota*. Moreover, lactate transport inhibition led to a notable expansion of *Bacteroidota* and *Actinomycetota*, while reducing the relative abundance of *Campylobacterota, Pseudomonadota*, and *Cyanobacteria* in ACLF rats (Figure 5A). At the genus level, compared with the Control group, ACLF rats showed a significant reduction in the abundance of *Eubacterium, Streptococcus, Bacteroides*, and *Ruminococcus*, while *Eubacterium, Hoylesella, Bifidobacterium*, and *Faecalibaculum* were markedly increased. Moreover, inhibition of lactate transport reduced the abundance of *Murimonas, Lachnospiraceae_NK4A136_group*, and *Terrisporobacter*, while increasing *Eubacterium, Ruminococcus*, and *Muribaculaceae* in ACLF rats (Figure 5B).

In addition, we observed that, at the phylum level, *Bacillota, Bacteroidota*, and *Actinomycetota* were the dominant taxa in the Control, ACLF, and ACLF+AR-C groups (Figure 5C). At the genus level, *Lactobacillus, Ligilactobacillus* and *Clostridium* were predominant in both the ACLF and ACLF+AR-C groups, whereas *Blautia* was the dominant genus in the Control group (Figure 5D). Additionally, LEfSe analysis showed that CK group showed higher LDA scores in *s_Ruminococcus_sp, o_Bacteroidales, c_Bacteroidia*, and *p_Bacteroidota*. However, ACLF group showed higher LDA scores in *s_Clostridium_disporicum, o_Eubacteriales, s_Lactobacillus_intestinalis*, and *s_Lactobacillus_oris*, ARC group showed higher LDA scores in *g_Lactobacillus* and *s_Lactobacillus_acidophilus* (Figures 5E, F).

Furthermore, PICRUSt2 was utilized to predict the functional capacities of the gut microbiota. The KEGG pathway annotations revealed that the identified pathways were predominantly related to nucleoside and nucleotide biosynthesis, amino acid biosynthesis, cofactor, carrier, and vitamin biosynthesis, fatty acid and lipid biosynthesis, carbohydrate degradation and cell structure biosynthesis (Supplementary Figure S2).

These results indicated that the gut microbiota structure in ACLF rats was substantially disrupted, with a marked expansion of *Lactobacillus* and related taxa, suggesting a potential association between *Lactobacillus* overgrowth and the development of ACLF. Moreover, inhibition of lactate transport further enhanced the abundance of *Lactobacillus* in the gut of ACLF rats.

### 3.4 Lactic acid levels were elevated in the serum, liver and feces of ACLF rats

**Figure 6:**
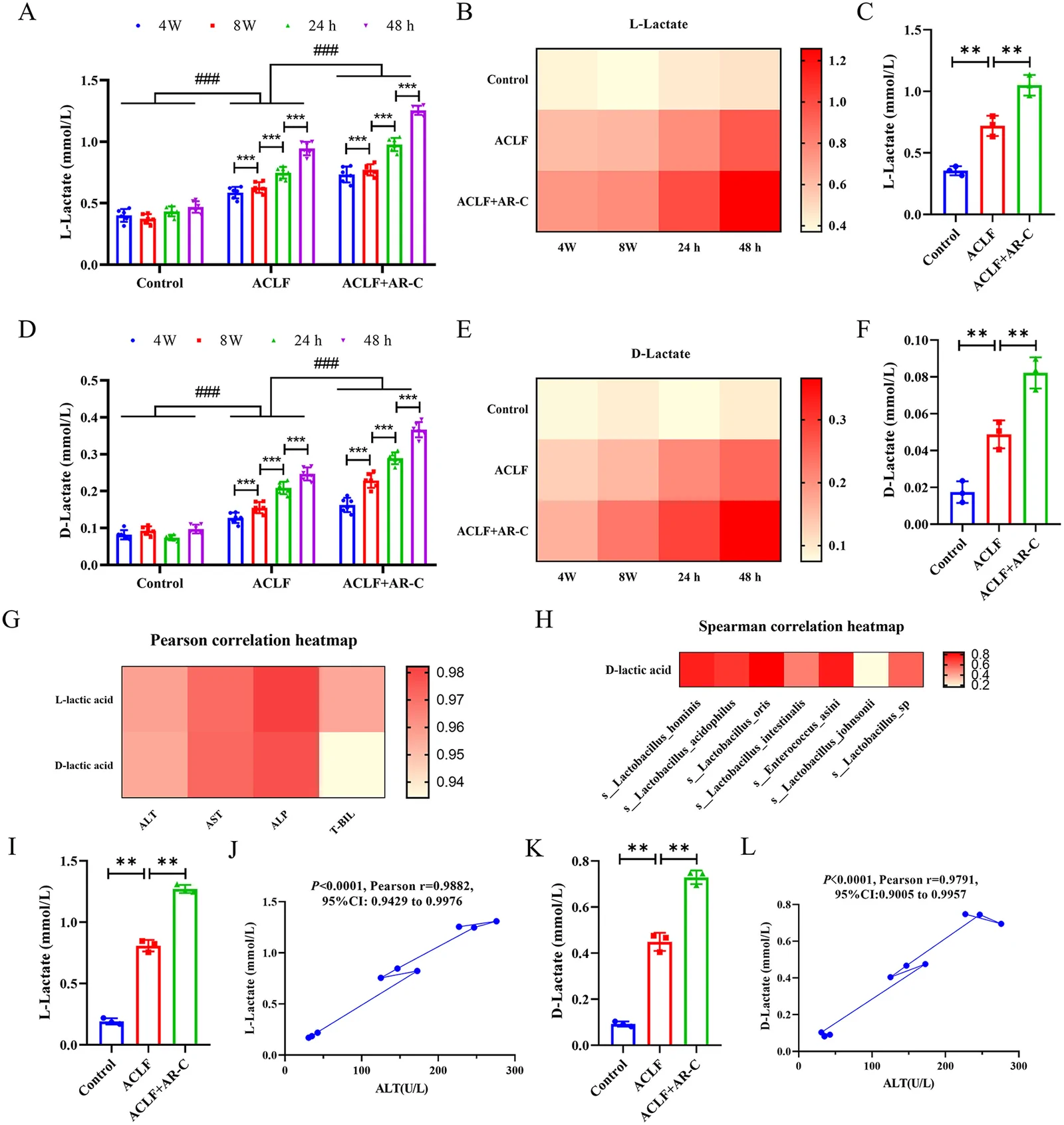
Detection of lactic acid content in rats. **(A)** Measurement of L-lactate levels in serum. *n* = 6 biological replicates per group. **(B)** Heatmap of serum L-lactate levels. *n* = 6 biological replicates per group. **(C)** Measurement of L-lactate levels in liver tissue. *n* = 3 biological replicates per group. **(D)** Measurement of D-lactate levels in serum. *n* = 6 biological replicates per group. **(E)** Heatmap of serum D-lactate levels. *n* = 6 biological replicates per group. **(F)** Measurement of D-lactate levels in liver tissue. *n* = 3 biological replicates per group. **(G)** Pearson correlation analysis between serum L-lactic acid and D-lactic acid levels and serum ALT, AST, ALP, and T-BIL levels in rats. **(H)** Spearman correlation analysis between serum D-lactic acid levels and the abundance of lactate-producing bacteria in rats. **(I)** Measurement of L-lactate levels in feces. *n* = 3 biological replicates per group. **(J)** Pearson correlation analysis between fecal L-lactate levels and serum ALT levels in rats. **(K)** Measurement of D-lactate levels in feces. *n* = 3 biological replicates per group. **(L)** Pearson correlation analysis between fecal D-lactate levels and serum ALT levels in rats. Comparisons among the three groups were performed using one-way analysis of variance (ANOVA), followed by Tukey's *post hoc* multiple-comparisons test. **P < 0.01, ***P < 0.001, ^###^*P* < 0.001.

As shown in Figures 6A–F, serum L-lactate and D-lactate levels in the ACLF group were significantly higher than those in the Control group at weeks 4 and 8 after model induction (*P* < 0.01). Moreover, serum L-lactate and D-lactate levels were significantly increased at 24 and 48 h in rats after acute onset (*P* < 0.01). Notably, the administration of the lactic acid transporter inhibitor in the ACLF group led to a further significant increase in these markers (*P* < 0.01). Similarly, in liver tissue, ACLF rats displayed significantly higher levels of L-lactate and D-lactate after acute onset compared to the Control group (*P* < 0.01), and lactic acid transporter inhibition further elevated these levels (*P* < 0.01). These results revealed that lactic acid levels were significantly elevated in the serum and liver after ACLF in rats. Moreover, lactic acid transporter inhibition could further increase the content of L-lactate and D-lactate in the serum and liver of ACLF rats.

Additionally, we analyzed the correlations between serum lactic acid levels and liver injury, between serum D-lactate levels and lactate-producing bacteria, and between fecal lactic acid levels and liver injury. Correlation analysis revealed a significant positive correlation between serum L-lactate levels and ALT (*r* = 0.958), AST (*r* = 0.971), ALP (*r* = 0.982), and T-BIL (*r* = 0.956; *P* < 0.01). Similarly, serum D-lactate level was also significantly positively correlated with ALT (*r* = 0.956), AST (*r* = 0.972), ALP (*r* = 0.978), and T-BIL (*r* = 0.934; *P* < 0.01; Figure 6G), suggesting that elevated serum L-lactate and D-lactate levels may be closely associated with the extent of liver injury. Further analysis showed that serum D-lactate level was significantly positively correlated with various lactate-producing bacteria, with the highest correlations observed for *Lactobacillus oris* (*r* = 0.8422), *Enterococcus asini* (*r* = 0.7717), and *Lactobacillus hominis* (*r* = 0.7584). Additionally, *Lactobacillus acidophilus* (*r* = 0.6994), *Lactobacillus* sp. (*r* = 0.5764), and *Lactobacillus johnsonii* (*r* = 0.1642) were also positively correlated with serum D-lactate (Figure 6H). Furthermore, fecal levels of L-lactate and D-lactate were significantly elevated in ACLF rats compared with Control rats (*P* < 0.01). Correlation analysis further showed that fecal L-lactate (*r* = 0.9882) and D-lactate (*r* = 0.9791) levels were significantly positively associated with serum ALT levels, indicating a close relationship between fecal lactate accumulation and liver injury (*P* < 0.01; Figures 6I–L).

These findings demonstrated that elevated serum D-lactic acid may be driven by an increase in the abundance of specific lactate-producing bacteria, and that changes in the gut microbiota may play a critical role in D-lactate production and the progression of liver injury. In conclusion, lactic acid levels were elevated in the serum, liver and feces of ACLF rats.

### 3.5 Expression of lactic acid transport-related proteins were inhibited in ACLF rats

**Figure 7:**
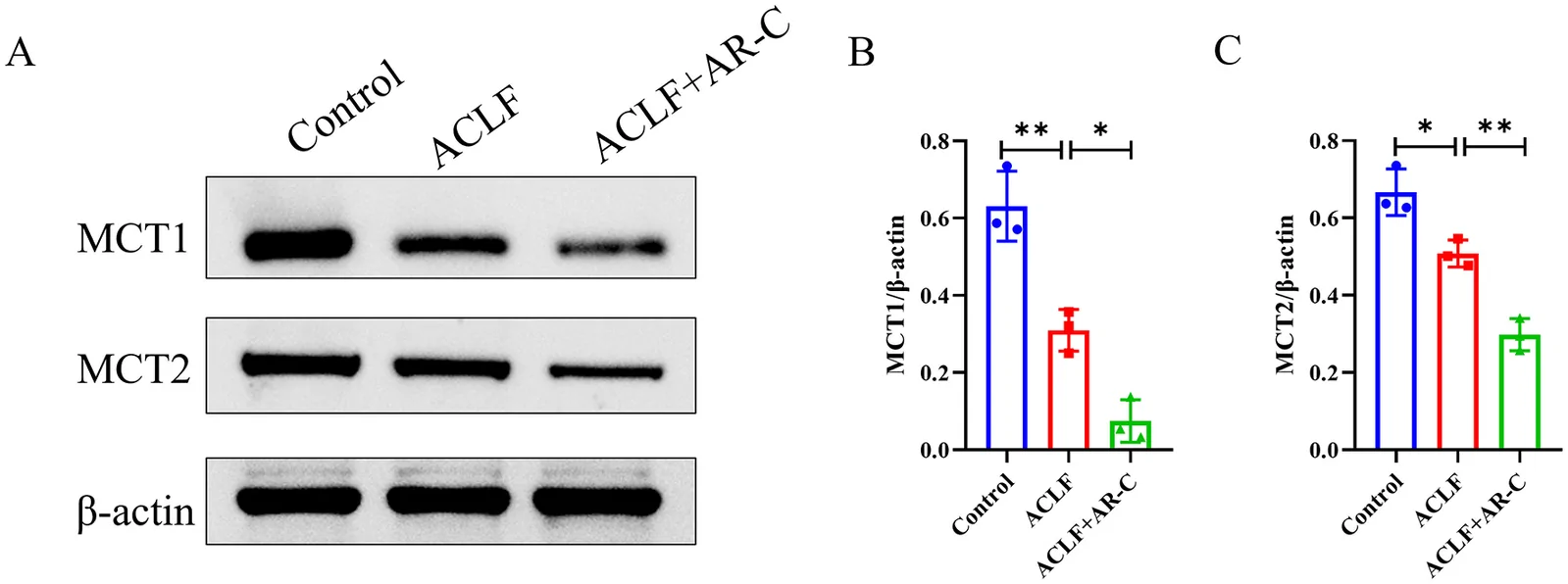
Lactic acid transporter-related proteins were detected by Western blot in liver tissue of rats. **(A)** Representative Western blot images of MCT1 and MCT2 proteins. **(B)** Quantification of relative MCT1 protein expression. **(C)** Quantification of relative MCT2 protein expression. *n* = 3 biological replicates per group. Comparisons among the three groups were performed using one-way analysis of variance (ANOVA), followed by Tukey's *post hoc* multiple-comparisons test. *P < 0.05, **P < 0.01.

WB analysis was performed to assess the expression of lactate transporter-related proteins MCT1 and MCT2 in liver tissue from each group of rats. The results showed that, compared with the Control group, the ACLF group exhibited a significant decrease in the expression levels of both MCT1 and MCT2 proteins (*P* < 0.01). Furthermore, the addition of the lactate transporter inhibitor to the ACLF group further significantly downregulated the expression of these proteins (*P* < 0.01; Figure 7). These results indicated that the expression of lactic acid transporter-related proteins were inhibited in ACLF rats. Moreover, the lactic acid transporter inhibitor further downregulated the expression of these proteins in ACLF rats.

### 3.6 Gut microbiota dysbiosis exacerbated the disruption of the gut microbiota composition in ACLF rats

**Figure 8:**
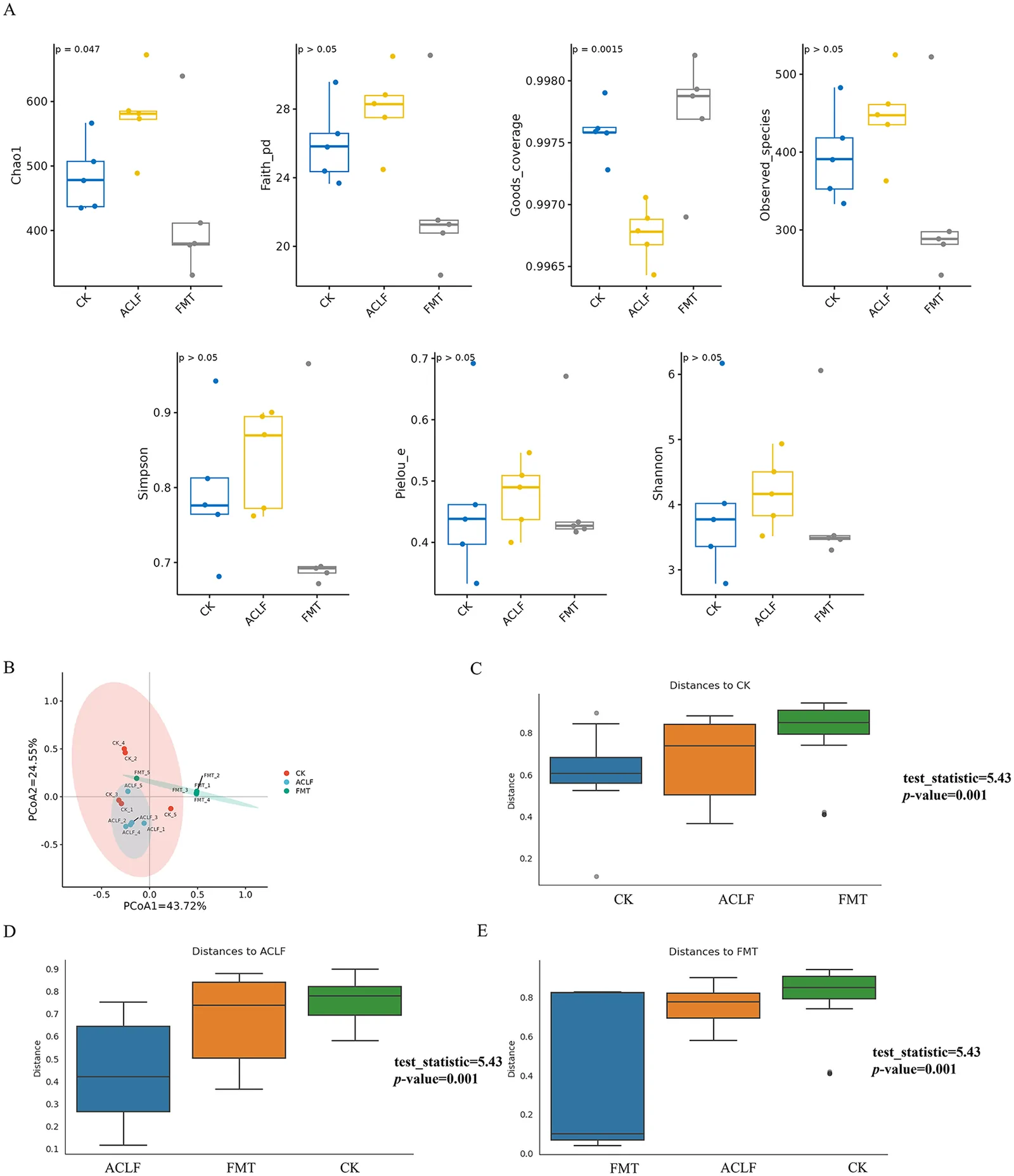
Analysis of gut microbial diversity. **(A)** Comparison of gut microbial α-diversity among the different groups. **(B)** PCoA of the gut microbiota based on Bray-Curtis distance. Statistical significance among groups was assessed using PERMANOVA/pseudo-F test based on Bray-Curtis distance. **(C)** β-diversity distances to the CK group based on Bray-Curtis dissimilarity. **(D)** β-diversity distances to the ACLF group based on Bray-Curtis dissimilarity. **(E)** β-diversity distances to the FMT group based on Bray-Curtis dissimilarity. *n* = 5 biological replicates per group. Differences in α-diversity indices among groups were assessed using the Kruskal-Wallis test followed by Dunn's multiple-comparisons test. Differences in β-diversity among groups were evaluated using permutational multivariate analysis of variance (PERMANOVA) based on Bray-Curtis dissimilarity, with the pseudo-F statistic used to assess statistical significance.

To investigate whether gut microbiota disorder would exacerbate liver injury in ACLF, we transplanted fecal contents from conventional ACLF rats into pseudo-germ-free ACLF rats. Next, 16S rRNA sequencing was used to examine the impact of the gut microbiota from conventional ACLF rats on the intestinal microbial profile of ACLF rats. Analysis of microbial taxonomic units showed that, at the phylum level, an average of 11 phyla were detected in the PG group, 12 phyla in the PG+ACLF group, and 11 phyla in the PG+ACLF+FMT group. At the genus level, an average of 139 genera were identified in both the PG and PG+ACLF groups, whereas only 119 genera were detected in the PG+ACLF+FMT group (Supplementary Table S4). Moreover, α-diversity measurements showed that compared with the PG+ACLF group, rats in the PG+ACLF+FMT group showed trends toward lower Chao1, Faith's phylogenetic diversity, observed species, Simpson, Pielou's evenness and Shannon indices, together with higher Good's coverage; however, none of these differences reached statistical significance (Figure 8A). Supplementary Figure S3 shows the rarefaction curve, species accumulation curve, and rank abundance curve. PCoA analysis revealed clear separation among the PG, PG+ACLF, and PG+ACLF+FMT groups (Figures 8B–E), indicating distinct microbial community structures across treatments.

**Figure 9:**
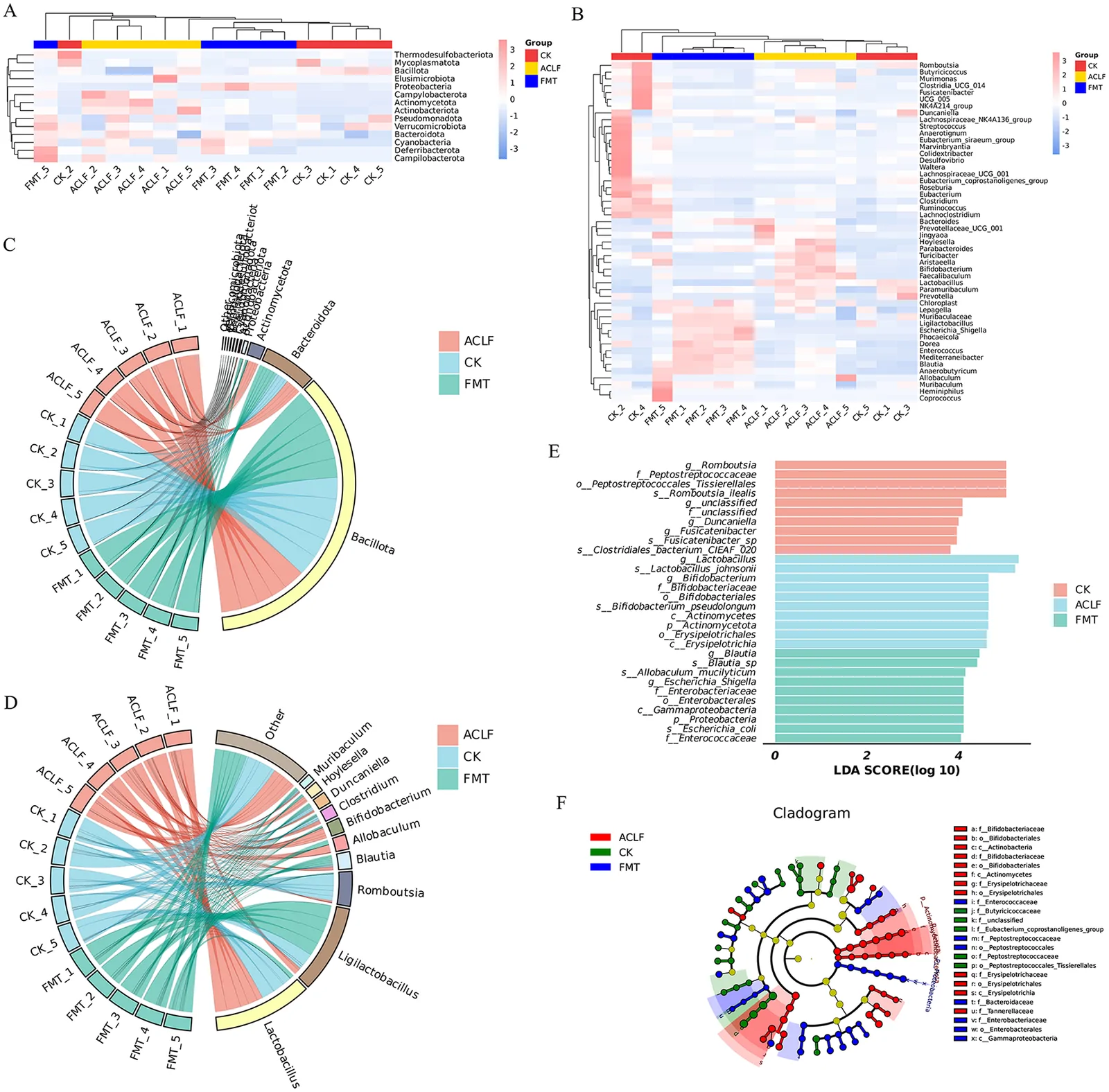
Differences in gut microbial composition and taxonomic biomarkers among groups. **(A)** Heatmap of differential bacterial abundance at the phylum level. **(B)** Heatmap of differential bacterial abundance at the genus level. **(C)** Analysis of dominant bacterial taxa at the phylum level. **(D)** Analysis of dominant bacterial taxa at the genus level. **(E)** LDA score bar plot. **(F)** Cladogram showing the taxonomic classification of the identified species. CK, Pseudo-germ-free group; ACLF, Pseudo-germ-free + ACLF model group; FMT, Pseudo-germ-free + ACLF + fecal microbiota transplantation group. FMT group refers to pseudo-germ-free ACLF rats receiving fecal microbiota transplantation from conventionally raised ACLF model rats. *n* = 5 biological replicates per group.

Additionally, we found that, at the phylum level, the PG+ACLF+FMT group exhibited a significant increase in the abundance of *Proteobacteria* compared with the PG+ACLF group, whereas the abundances of *Campylobacterota, Actinomycetota, Actinobacteriota*, and *Mycoplasmatota* were markedly reduced (Figure 9A). At the genus level, the PG+ACLF+FMT group showed significantly higher abundances of *Muribaculaceae, Ligilactobacillus, Escherichia-Shigella, Mediterraneibacter*, and *Blautia* relative to the PG+ACLF group, while *Parabacteroides, Turicibacter, Aristaeella, Bifidobacterium*, and *Faecalibaculum* were significantly decreased (Figure 9B).

In addition, we observed that *Bacillota* dominated the gut microbiota at the phylum level in both the PG+ACLF and PG+ACLF+FMT groups (Figure 9C). At the genus level, *Lactobacillus* was the dominant taxon in the PG+ACLF group, whereas *Ligilactobacillus* predominated in the PG+ACLF+FMT group (Figure 9D). LDA score analysis confirmed that *Escherichia_coli* and *Enterococcaceae* exhibited the highest discriminative scores in the PG+ACLF group. LDA score analysis revealed that lactate-producing taxa, such as *Escherichia coli* and *Enterococcaceae*, were significantly enriched in the PG+ACLF+FMT group (Figures 9E, F). PICRUSt2 analysis showed that the identified pathways were predominantly related to nucleoside and nucleotide biosynthesis, amino acid biosynthesis, carbohydrate degradation, cofactor, carrier, and vitamin biosynthesis, fatty acid and lipid biosynthesis, cell structure biosynthesis and carbohydrate biosynthesis (Supplementary Figure S4).

### 3.7 Gut microbiota dysbiosis increased the lactate levels in ACLF rats

**Figure 10:**
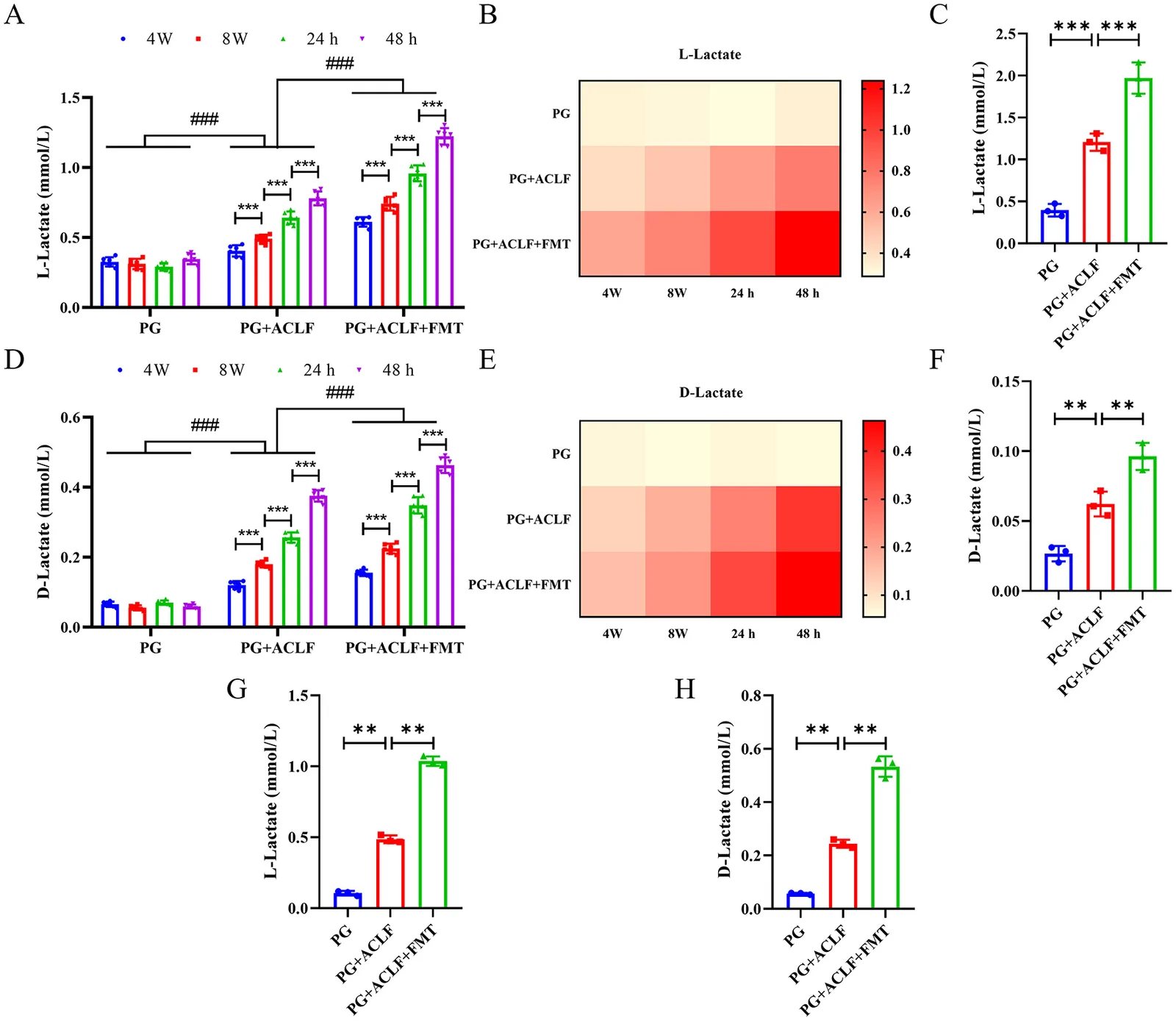
Gut microbiota dysbiosis increased the lactic acid levels in ACLF rats. **(A)** Measurement of L-lactate levels in serum. *n* = 6 biological replicates per group. **(B)** Heatmap of serum L-lactate levels. *n* = 6 biological replicates per group **(C)** Measurement of L-lactate levels in liver tissue. *n* = 3 biological replicates per group. **(D)** Measurement of D-lactate levels in serum. *n* = 6 biological replicates per group. **(E)** Heatmap of serum D-lactate levels. *n* = 6 biological replicates per group. **(F)** Measurement of D-lactate levels in liver tissue. *n* = 3 biological replicates per group. **(G)** Measurement of L-lactate levels in feces. *n* = 3 biological replicates per group. **(H)** Measurement of D-lactate levels in feces. *n* = 3 biological replicates per group. Comparisons among the three groups were performed using one-way analysis of variance (ANOVA), followed by Tukey's *post hoc* multiple-comparisons test. **P < 0.01, ***P < 0.001, ^###^*P* < 0.001.

Figures 10A–F showed that serum levels of L-lactate and D-lactate in the PG+ACLF group significantly increased at the 4th and 8th weeks after model inducing, and at 24 h and 48 h after acute onset compared with the PG group (*P* < 0.05). Moreover, relative to the PG+ACLF group, the PG+ACLF+FMT group exhibited further significant elevations in serum L-lactate and D-lactate at all corresponding time points (*P* < 0.01). In liver tissue, L-lactate and D-lactate levels were markedly higher in the PG+ACLF group than in the PG group (*P* < 0.01), and these levels were further significantly increased in the PG+ACLF+FMT group (*P* < 0.01). Additionally, our results showed that fecal microbiota transplantation also significantly increased fecal L-lactate and D-lactate levels in rats in the PG+ACLF group (*P* < 0.01; Figures 10G, H). Collectively, these findings indicated that gut microbiota dysbiosis could further elevate lactate levels in ACLF rats.

### 3.8 Gut microbiota dysbiosis exacerbated liver injury and systemic inflammation in ACLF rats

**Figure 11:**
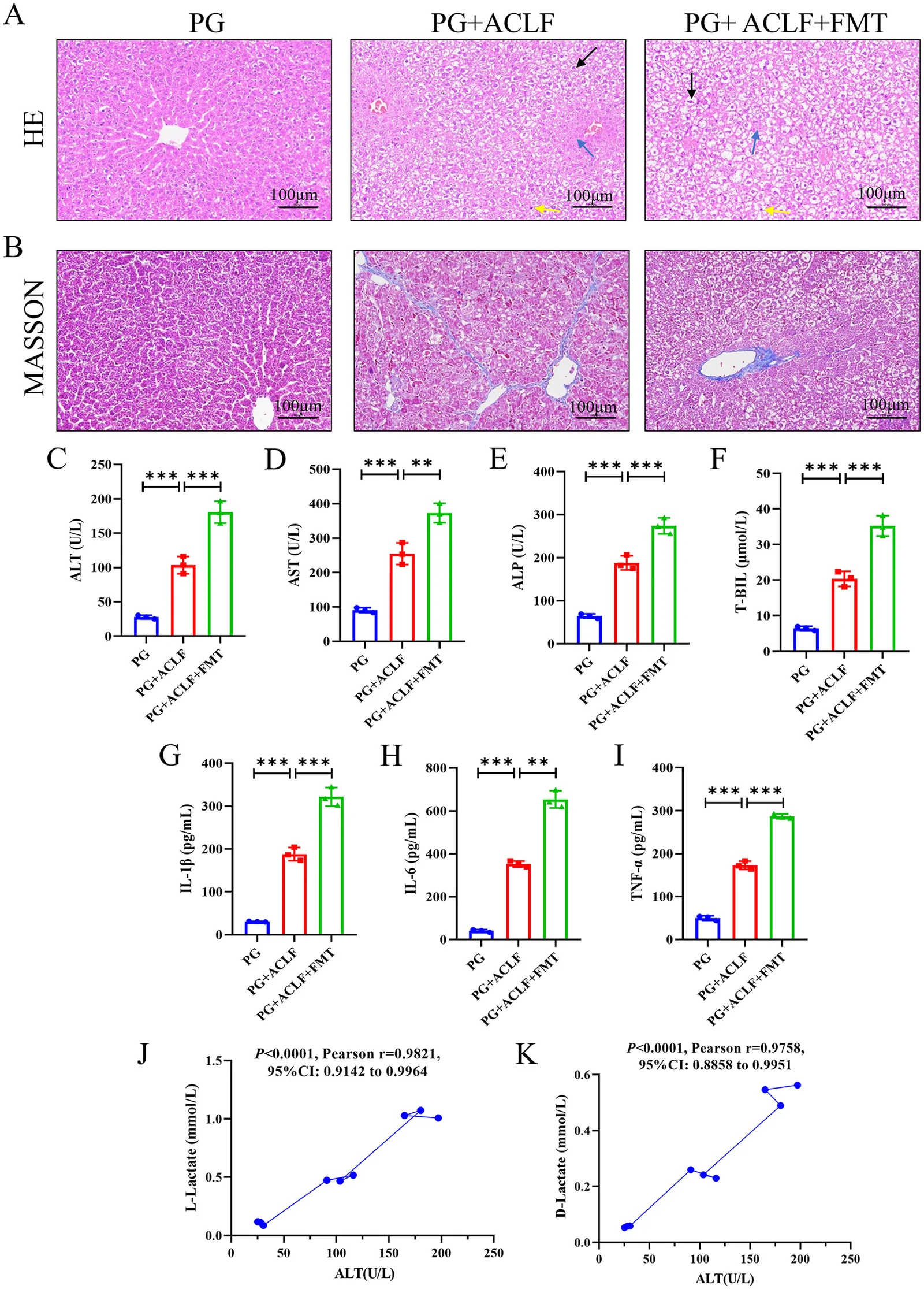
Gut microbiota dysbiosis exacerbated liver injury and systemic inflammation in ACLF rats. **(A)** HE staining was used to observe histopathological alterations. **(B)** Masson staining was used to evaluate hepatic fibrosis. **(C–I)** Serum levels of ALT **(C)**, AST **(D)**, ALP **(E)**, T-BIL **(F)**, IL-1β **(G)**, IL-6 **(H)**, and TNF-α **(I)** in rats. **(J)** Pearson correlation analysis between fecal L-lactate levels and serum ALT levels in rats. **(K)** Pearson correlation analysis between fecal D-lactate levels and serum ALT levels in rats. *n* = 3 biological replicates per group. Comparisons among the three groups were performed using one-way analysis of variance (ANOVA), followed by Tukey's *post hoc* multiple-comparisons test. **P < 0.01, ***P < 0.001.

HE staining (Figure 11A) showed that liver tissues from the PG group exhibited intact architecture, with uniformly distributed hepatic lobules, well-organized hepatic cords, and regularly shaped hepatocytes, without apparent pathological alterations. In the PG+ACLF group, hepatic cords were disorganized, hepatocytes were irregularly arranged with diffuse cell injury and indistinct cell borders, and marked connective tissue proliferation with mild inflammatory infiltration was observed in the portal area. In the PG+ACLF+FMT group, hepatic cords were similarly disrupted, hepatocyte arrangement was disordered, and extensive hepatocellular swelling with cytoplasmic rarefaction was noted, accompanied by mild inflammatory infiltration. Masson staining (Figure 11B) revealed normal liver morphology without evident fibrosis in the PG group, whereas both the PG+ACLF and PG+ACLF+FMT groups exhibited marked fibrosis around hepatic sinusoids.

Serum biochemical analyses (Figures 11C–F) showed that ALT, AST, ALP, and T-BIL levels were significantly elevated in the PG+ACLF group compared with the PG group at 48 h after acute attack (*P* < 0.01). Notably, transplantation of fecal contents from conventional ACLF rats into pseudo-germ-free ACLF rats significantly increased these serum markers at 48 h after acute attack (*P* < 0.01). Notably, fecal microbiota transplantation also significantly increased the serum levels of IL-1β, IL-6 and TNF-α in rats in the PG+ACLF group at 48 h after acute attack (*P* < 0.01; Figures 11G–I). Moreover, fecal L-lactate (*r* = 0.9821) and D-lactate (*r* = 0.9758) levels were significantly positively correlated with serum ALT levels in rats (Figures 11J, K).

Collectively, these findings indicated that exacerbation of gut microbial dysbiosis may promote liver injury in ACLF rats.

## 4 Discussion

ACLF is a critical clinical syndrome in which an acute liver failure occurs on the background of chronic liver disease, leading to multiple organ dysfunction syndrome and high short-term mortality. To the best of our knowledge, compared with patients with other forms of chronic liver failure, such as cirrhosis, patients with ACLF usually exhibit significantly higher serum levels of ALT, AST, and TBIL (Tang et al., 2023; Zhao et al., 2022). In agreement, our findings also demonstrated that serum ALT, AST, ALP, and TBIL levels were significantly elevated in patients with ACLF relative to those with other chronic liver failure patients. According to recent studies, ALT and AST are intracellular enzymes released into the circulation following hepatocellular damage, reflecting the extent of hepatocyte necrosis and inflammation (Xuan et al., 2024). ALP is primarily elevated in conditions involving cholestasis and biliary obstruction, indicating impaired bile flow (Zhang et al., 2023). TBIL reflects the liver's capacity for bilirubin uptake, conjugation, and excretion, and increased levels signify severe hepatocellular dysfunction or cholestasis. Collectively, these results indicated that patients with ACLF may experience more severe liver injury than those with other forms of chronic liver failure. Moreover, we also found that patients with ACLF exhibited significantly higher serum levels of both L-lactate and D-lactate. Elevated lactic acid levels closely correlate with the severity of liver injury, systemic inflammation and poor clinical outcomes, and are widely used as a prognostic indicator in liver failure (Gao et al., 2019; Yao et al., 2024). Similarly, we found that serum L-lactate and D-lactate levels in patients with ACLF were significantly positively correlated with ALT levels, a marker of liver injury. Therefore, we speculated that elevated lactic acids may be positively associated with severe liver injury in patients with ACLF.

Here, we have established a rat model of ACLF and administered AR-C155858, a lactic acid transport inhibitor, to ACLF rats to assess the impact of blocking lactate translocation on liver injury. AR-C155858 is a potent inhibitor of monocarboxylate transporters MCT1 and MCT2 (Guan et al., 2019; Ovens et al., 2010). It binds to an intracellular site within MCT1 and inhibits proton-coupled, bidirectional transport of lactate and other monocarboxylates across the cell membrane. Consequently, AR-C155858 impairs cellular lactate uptake and efflux, thereby disrupting lactate transport and systemic lactate homeostasis. As expected, inhibition of lactic acid transport significantly exacerbated liver injury and systemic inflammation in ACLF rats. The liver is the principal organ responsible for systemic lactic acid clearance (Wang et al., 2025). Previous work has reported that increased lactic acid levels can be frequently found in liver disease, particularly among patients with chronic liver disease (Scheiner et al., 2017). Additionally, a comparison of 28-day survival in patients with cirrhosis revealed that survivors had significantly lower admission lactic acid levels than non-survivors, and lactic acid clearance was markedly higher in survivors (Drolz et al., 2019). Mechanistically, elevated circulating lactic acid can aggravate liver damage, potentially through promoting hepatocyte ferroptosis via enhanced phosphoenolpyruvate carboxykinase 2 (PCK2) activity (Yuan et al., 2025). Moreover, lactic acid has been reported to promote hepatic stellate cell activation and lactylation, thereby accelerating the progression of liver fibrosis (Yao et al., 2024). Importantly, fibrosis-induced structural remodeling and matrix deposition usually exacerbate the damage of liver function (Dulai et al., 2017). Collectively, our findings indicated that inhibition of lactic acid transport may aggravate liver injury in ACLF, supporting a pathogenic role for lactic acid accumulation in ACLF progression.

Previous study uncovered that gut microbial diversity and abundance significantly declined during the progression of ACLF (Yao et al., 2021). Meanwhile, the relative abundances of potentially pathogenic genera, such as *Klebsiella, Veillonella, Streptococcus*, and *Enterococcus*, were significantly increased. Another related study also reported that compared with patients with other chronic liver failure and healthy individuals, ACLF patients exhibited a significant reduction in gut microbiota diversity and abundance, and the abundances of *Lactobacillus casei paracasei, Enterococcus faecium*, and *Enterococcus* increased progressively with disease severity (Wang K. et al., 2021). These studies suggested that the progression of ACLF may be closely associated with alterations in the gut microbiota. Here, our results showed a trend toward reduced gut microbial diversity in ACLF rats, although most α-diversity differences did not reach statistical significance, together with distinct alterations in microbial community composition. Detailedly, *Actinomycetota* exhibited the most prominent increase, whereas *Bacteroidota* showed the largest decrease in ACLF rats. Notably, inhibition of lactic acid transport further enhanced the abundance of *Actinomycetota*. At the genus level, the abundances of *Eubacterium, Streptococcus, Bacteroides* and *Ruminococcus* were significantly reduced, while *Eubacterium, Hoylesella, Bifidobacterium*, and *Faecalibaculum* were markedly enriched in ACLF rats. Moreover, blocking lactic acid transport decreased the abundances of *Murimonas, Lachnospiraceae_NK4A136_group*, and *Terrisporobacter*, while increasing *Eubacterium, Ruminococcus*, and *Muribaculaceae* in ACLF rats. Moreover, we found that *Lactobacillus, Ligilactobacillus* and *Clostridium* were dominant taxa in the gut of ACLF rats, and their abundances, particularly *Lactobacillus* and *Lactobacillus acidophilus*, were further elevated following lactic acid transport inhibition.

In the intestinal environment, *Lactobacillus* produces lactic acid through the fermentation of dietary carbohydrates (Wang Y. et al., 2021). Importantly, microbial metabolites generated in the gut, such as lactic acid, can cross the intestinal barrier and enter the systemic circulation (Caspani et al., 2019; Remund et al., 2023). Therefore, we speculated that the increased abundance of lactate-producing bacteria in ACLF rats may elevate serum lactic acid levels in ACLF rats. Consistently, our results found that D-lactate and L-lactate concentrations were significantly elevated in serum, liver tissues and feces in ACLF rats. Notably, D-lactate and L-lactate are two stereoisomers of lactate with distinct biological sources and pathophysiological implications (Pohanka, 2020). L-lactate is the predominant lactate stereoisomer in mammalian tissues and is mainly produced from pyruvate by lactate dehydrogenase during glycolysis, particularly under hypoxic, inflammatory or metabolically stressed conditions. In contrast, D-lactate is produced only in trace amounts in mammalian tissues, mainly through the methylglyoxal–glyoxalase pathway, whereas a substantial increase in D-lactate is generally associated with intestinal bacterial fermentation. Here, we found that serum D-lactate level in ACLF rats were significantly positively correlated with the abundance of lactate-producing bacteria, including *Lactobacillus oris, Enterococcus asini, Lactobacillus hominis, Lactobacillus acidophilus, Lactobacillus johnsonii*, and *Lactobacillus* sp. Therefore, in the present study, elevated D-lactate levels may more directly reflect gut microbiota-derived lactate production, whereas increased L-lactate level may represent the combined effects of host metabolic dysfunction, inflammatory stress and microbial fermentation during ACLF progression. Moreover, our results showed that serum and fecal D-lactic acid levels were significantly positively correlated with serum liver injury markers. In conclusion, our results indicated that the increased abundance of *Lactobacillus* in the gut of ACLF rats could contribute to elevated D-lactate level in serum and increased serum D-lactate level may aggravate the impairment of liver function impairment in ACLF rats.

Further, we investigated whether gut microbiota derived from ACLF rats could exacerbate liver injury in ACLF rats. Firstly, we examined the effects of fecal microbiota transplantation on gut microbial diversity and compositional profiles in ACLF rats. Here, we found that gut microbiota derived from conventional ACLF rats reduced microbial diversity and altered the community structure in pseudo-germ-free ACLF rats. In addition, fecal microbiota transplantation increased the abundance of lactate-producing taxa, such as *Ligilactobacillus, Enterococcus* and *Escherichia-Shigella*. Results from the literature showed that members of the genus *Ligilactobacillus* are homofermentative lactic acid bacteria that produce lactic acid as the major end product of carbohydrate fermentation (Chuandong et al., 2024). Moreover, *Enterococcus* is recognized as a homofermentative lactic acid bacterium capable of efficiently converting carbohydrates into lactic acid and is widely utilized in lactic acid fermentation (Subramanian et al., 2015). Members of the genus *Escherichia-Shigella*, such as *Escherichia coli*, perform mixed-acid fermentation under anaerobic conditions, generating lactic acid (Vuoristo et al., 2015). Collectively, gut microbiota derived from conventional ACLF rats could increase the abundance of lactate-producing bacteria in the intestines of pseudo-germ-free ACLF rats. These results may be attributable to the intrinsically high abundance of lactate-producing bacteria, such as *Lactobacillus* and *Ligilactobacillus*, in the gut microbiota of conventional ACLF rats. Fecal microbiota transplantation likely further increased the abundance of these lactate-producing taxa in the intestines of pseudo-germ-free ACLF rats.

In addition, we also observed that transplantation of fecal microbiota from conventional ACLF rats increased the abundance of liver injury-associated taxa in pseudo-germ-free ACLF rats, such as *Enterococcus* and *Escherichia-Shigella*. Previous study has reported that *Enterococcus faecalis* and *Escherichia coli* were significantly enriched in the gut microbiota of patients with metabolic dysfunction-associated steatotic liver disease (MASLD) and may induce hepatocellular damage by secreting exotoxin (Beyoglu and Idle, 2025). Therefore, we speculated that fecal microbiota transplantation could increase lactic acid levels in pseudo-germ-free ACLF rats, thereby exacerbating liver injury. In agreement, lactic acid quantification also demonstrated significantly higher D-lactate and L-lactate concentrations in both the serum, liver tissues and feces of pseudo-germ-free ACLF rats. Additionally, we also demonstrated that fecal microbiota transplantation markedly exacerbated liver injury and inflammation in pseudo-germ-free ACLF rats, accompanied by significantly elevated serum ALT, AST, ALP, T-BIL IL-1β, IL-6, and TNF-α. These results indicated that gut microbiota derived from conventional ACLF rats could aggravate liver injury in pseudo-germ-free ACLF rats by increasing the content of D-lactate in serum and feces.

Several limitations of this study should be acknowledged. First, the relatively small sample size of the animal experiments resulted in limited statistical power, which may have reduced the ability to detect modest between-group differences. Therefore, the animal findings should be interpreted as exploratory and require confirmation in larger, adequately powered studies. Second, we did not directly evaluate changes in the gut microbiota before and after establishment of the pseudo-germ-free model. Although the modeling procedure was performed according to previously established protocols, the efficiency of microbiota depletion should ideally have been verified by fecal bacterial culture, 16S rRNA gene-based qPCR, or other quantitative approaches. Third, the FMT experiments did not include a healthy-donor FMT control group. Consequently, the observed effects cannot fully distinguish the specific contribution of ACLF-derived microbiota from the general effects of microbial recolonization following microbiota depletion. Fourth, the detailed molecular mechanisms linking microbial lactic acid to liver injury, including lactic acid transport signaling and downstream metabolic or epigenetic effects, remain to be defined. Fifth, whether direct inhibition of lactate-producing bacterial proliferation can reduce lactate accumulation and alleviate liver injury in ACLF remains to be determined. Taken together, these limitations restrict definitive causal interpretation of the present findings, and our results should therefore be considered as supporting a potential contribution of gut microbiota dysbiosis and lactate accumulation to ACLF-associated liver injury rather than establishing a definitive causal relationship.

## 5 Conclusion

**Figure 12:**
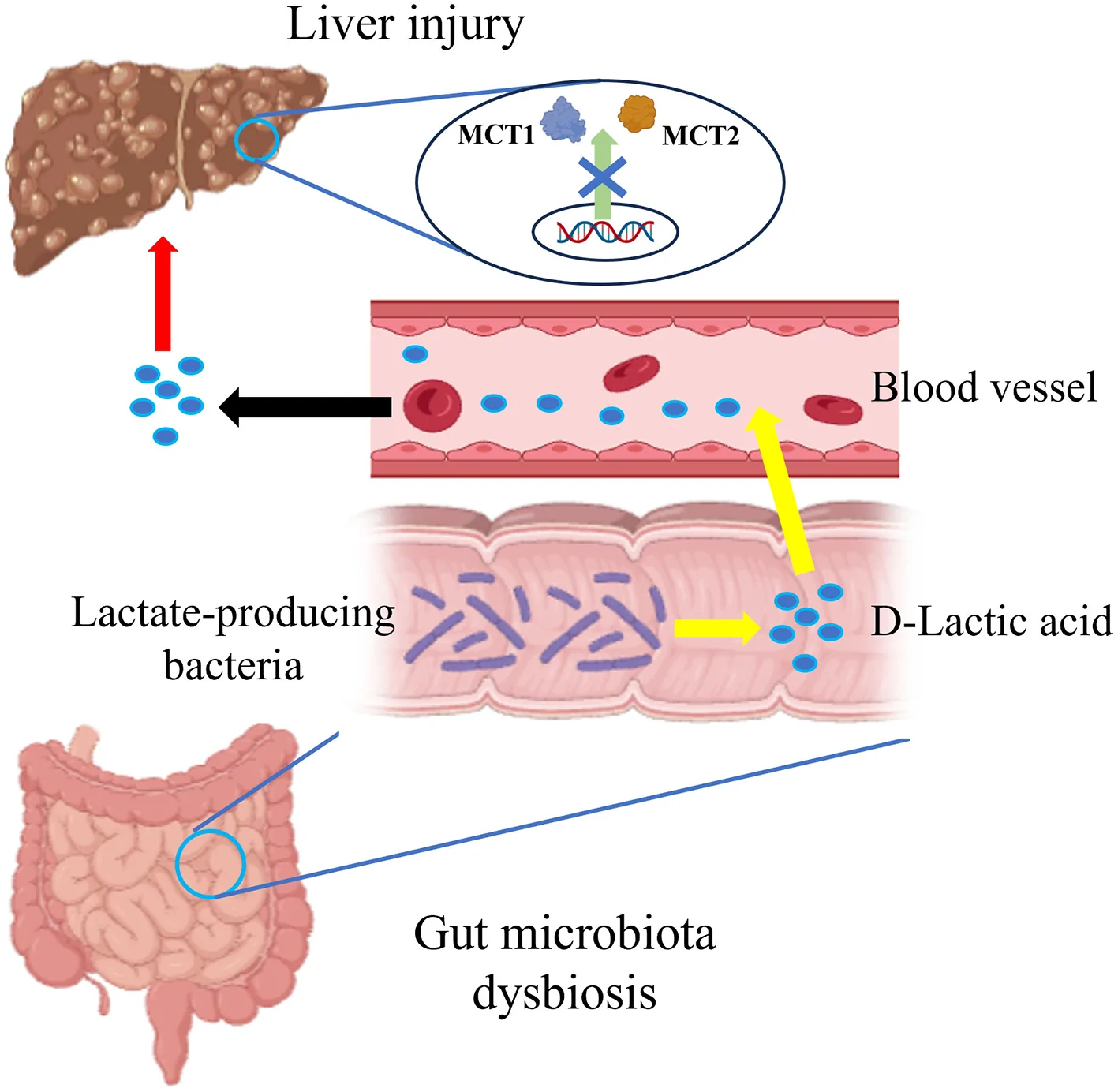
Molecular mechanism diagram of gut microbiota dysbiosis exacerbating liver injury. In patients with ACLF, the abundance of lactate-producing bacteria in the gut is increased, leading to elevated intestinal D-lactic acid levels. Excess lactate translocates across the intestinal barrier into the circulation and is subsequently delivered to the liver. In the setting of impaired hepatic function, lactate clearance and metabolism are reduced, resulting in hepatic lactate accumulation, which further exacerbates hepatocellular injury.

In summary, our study demonstrated that inhibition of lactic acid transport could exacerbate liver injury in ACLF rats. Moreover, the abundance of lactate-producing bacteria, such as *Lactobacillus*, was significantly elevated in the gut of ACLF rats, accompanied by a substantial increase in D-lactate and L-lactate levels in both serum, liver tissues and feces. Therefore, we speculated that the enrichment of lactate-producing bacteria in the gut would enhance systemic lactic acid accumulation, particularly D-lactate, thereby aggravating liver injury in ACLF rats (Figure 12). Our findings provided new insights into how gut microbiota dysbiosis would induce liver injury in patients with ACLF and establish a theoretical foundation for the future development of novel therapeutic strategies targeting ACLF.

## Data Availability

The data used in this study are available from the corresponding authors upon reasonable requests. The original 16S rRNA sequencing data used in this study was deposited in the Science Data Bank. The online version contains the RNA-seq data, available at: https://www.scidb.cn/en/s/iQNVni.

## Supplementary material

Supplementary Figure S1 Assessment of sequencing depth and community diversity in the gut microbiota. **(A)** Rarefaction curve. **(B)** Species accumulation curves. **(C)** Rank abundance curve.

Supplementary Figure S2 The function of microbial genes predicted by PICRUSt2 based on annotations from KEGG databases.

Supplementary Figure S3 Assessment of sequencing depth and community diversity in the gut microbiota. **(A)** Rarefaction curve. **(B)** Species accumulation curves. **(C)** Rank abundance curve.

Supplementary Figure S4 The function of microbial genes predicted by PICRUSt2 based on annotations from KEGG databases.
